# Edaravone Dexborneol mitigates pathology in animal and cell culture models of Alzheimer’s disease by inhibiting neuroinflammation and neuronal necroptosis

**DOI:** 10.1186/s13578-024-01230-8

**Published:** 2024-04-27

**Authors:** Chong Xu, Yilan Mei, Ruihan Yang, Qiudan Luo, Jienian Zhang, Xiaolin Kou, Jianfeng Hu, Yujie Wang, Yue Li, Rong Chen, Zhengping Zhang, Yuyuan Yao, Jian Sima

**Affiliations:** 1 Laboratory of Aging Neuroscience and Neuropharmacology, School of Basic Medicine and Clinical Pharmacy, China Pharmaceutical University, Nanjing, 210009 China; 2 Department of Pharmacology, NeuroDawn Pharmaceutical Co., Ltd, Nanjing, 211199 China

**Keywords:** Alzheimer’s disease, Edaravone Dexborneol, Astrocyte, Neuroinflammation, Necroptosis

## Abstract

**Background:**

Alzheimer’s disease (AD) is the most prevalent neurodegenerative disease with limited disease-modifying treatments. Drug repositioning strategy has now emerged as a promising approach for anti-AD drug discovery. Using 5×FAD mice and Aβ-treated neurons in culture, we tested the efficacy of Y-2, a compounded drug containing the antioxidant Edaravone (Eda), a pyrazolone and (+)-Borneol, an anti-inflammatory diterpenoid from cinnamon, approved for use in amyotrophic lateral sclerosis patients.

**Results:**

We examined effects of Y-2 versus Eda alone by *i.p.* administered in 8-week-old 5×FAD mice (females) for 4 months by comparing cognitive function, Aβ pathologies, neuronal necroptosis and neuroinflammation. Using primary neurons and astrocytes, as well as neuronal and astrocytic cell lines, we elucidated the molecular mechanisms of Y-2 by examining neuronal injury, astrocyte-mediated inflammation and necroptosis. Here, we find that Y-2 improves cognitive function in AD mice. Histopathological data show that Y-2, better than Eda alone, markedly ameliorates Aβ pathologies including Aβ burden, astrogliosis/microgliosis, and Tau phosphorylation. In addition, Y-2 reduces Aβ-induced neuronal injury including neurite damage, mitochondrial impairment, reactive oxygen species production and NAD^+^ depletion. Notably, Y-2 inhibits astrocyte-mediated neuroinflammation and attenuates TNF-α-triggered neuronal necroptosis in cell cultures and AD mice. RNA-seq further demonstrates that Y-2, compared to Eda, indeed upregulates anti-inflammation pathways in astrocytes.

**Conclusions:**

Our findings infer that Y-2, better than Eda alone, mitigates AD pathology and may provide a potential drug candidate for AD treatment.

**Supplementary Information:**

The online version contains supplementary material available at 10.1186/s13578-024-01230-8.

## Background

Alzheimer’s disease (AD), the most common neurodegenerative disease (ND), follows a progressive process and is featured by neuritic dystrophy, neuronal loss and cognitive impairment [1–4]. Amyloid-beta (Aβ) is known to play a key role in AD pathogenesis, which is confirmed by familial mutations of amyloid precursor protein (APP) and presenilin and defines the disease and vaccines against Aβ have shown therapeutic promise [5]. Although evidence is still accumulating, this hypothesis is inadequate to resolve many characteristics of AD pathophysiology. Therefore, other basic hallmarks associated with AD are currently gaining attention, including the formation of neurofibrillary tangles due to Tau aggregation, aberrant neuroinflammation, reduced NAD^+^ levels, and the oxidative damage of lipids, DNA, and proteins [6–9]. The anti-Aβ therapies, if combined with other basic hallmarks-modifying strategies, may thus have the potential to achieve more efficacy for AD.

Increasing evidence has demonstrated that Aβ-induced glial activation can further elevate the levels of proinflammatory cytokines, such as TNF-α and IL-6 [10], which leads to excessive or chronic neuroinflammation and may contribute to AD pathogenesis [11]. In addition to its proinflammatory function, excess TNF-α also triggers neuronal necroptosis, as another source of neuronal loss in AD brain, through activation of the receptor-interacting serine/threonine-protein kinase 1 and 3 (RIPK1/3) and the mixed lineage kinase domain-like (MLKL), which can be inhibited by intracerebral injection of TNF-α neutralizing antibody or by downregulation of SQSTM1 (p62) [12, 13]. As a highly redox-active agent, Aβ also triggers an overproduction of reactive oxygen species (ROS) [14], which in turn aggravates Aβ-induced neuronal damage and exacerbates the progression of AD [15]. Therefore, anti-oxidative stress is also an important strategy in the treatment of AD, and a few antioxidant drugs do show therapeutic promise [16, 17]. Studies have demonstrated the direct link between the reduction of nicotinamide adenine dinucleotide (NAD^+^) and cell senescence in AD brains. Also, orally administered with NAD^+^ precursors has shown promising therapeutic effects in various AD models [18, 19]. Transcription factor NF-κB regulates inflammation and redox in many systems including AD. In fact, activated p65, a subunit of NF-κB, has been observed in neurons and astrocytes surrounding Aβ plaques, and it may induce neuroinflammation as well as oxidative stress [20, 21]. Several phytochemicals, such as polyphenols and alkaloids have been reported as potent NF-κB inhibitors in AD models [22]. Therefore, treatments targeting multiple pathogenic signaling pathways, such as neuroinflammation, oxidative stress, and neuronal death, may be an important strategy for drug development against AD.

As a potent free radical scavenger, Edaravone (Eda) was first approved for the clinical treatment of acute ischemic stroke (AIS) and amyotrophic lateral sclerosis (ALS) in Japan [23, 24], and then approved by FDA for the treatment of ALS in US [25]. Recent evidence has also suggested neuroprotective effects of Eda in AD animal models by alleviating Aβ deposition, oxidative stress and other Aβ-associated pathologies [17, 26]. (+)-Borneol, a naturally occurring terpene and bicyclic organic compound, has been found to inhibit the production of inflammatory factors and protect brain function in preclinical studies [27, 28], possibly by targeting NF-κB (p65), nitric oxide synthase, and ICAM-1 [27, 29]. Recently, Y-2, an intravenous solution containing concentrated Eda and (+)-Borneol, has been approved for the treatment of AIS in China and Y-2 sublingual tablets, another Y-2 formulation containing 30 mg of Eda and 6 mg of (+)-Borneol per tablet, are undergoing a phase I clinical trial in US (www.clinicaltrials.gov, NCT03495206), and a phase III clinical trial in China for AIS treatment (www.chinadrugtrials.org.cn, CTR20210233) [30]. Notably, in patients with AIS, Y-2 has shown better neuroprotective potency compared to Eda alone [31], indicating a potential for neurological diseases. In this study, we have demonstrated a better efficacy of Y-2 compared to Eda alone, including cognitive improvement, and inhibition of Aβ pathologies, neuronal necroptosis and NF-κB-associated neuroinflammation. In addition, we use in vitro models of cultured neurons and astrocytes to evaluate the neuronal injury, astrocyte-mediated inflammation and necroptosis. Thus, our findings reveal that Y-2, as a high potential candidate, may provide a multi-targeted therapeutic drug for AD treatment.

## Results

### Y-2 ameliorates cognitive impairment of the 5×FAD mice

**Fig. 1:**
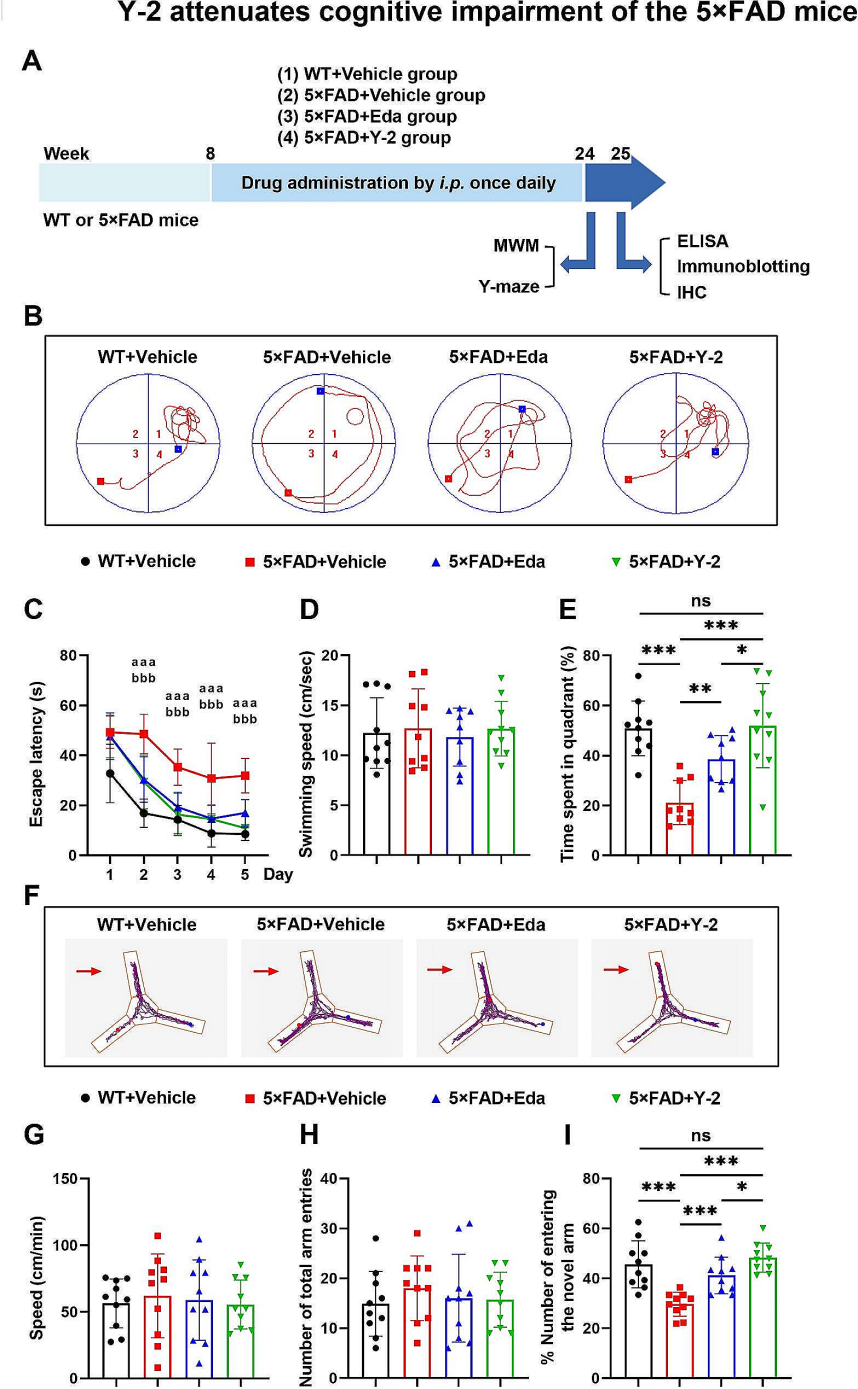
Y-2 attenuates cognitive impairment of the 5×FAD mice. (**A**) A schematic overview shows the animal experimental procedures. (**B**) Representative trajectories in MWM assays on the testing day 6. Red square, starting point; blue square, ending point; platform (red circle) locates in the quadrant 1. (**C**) Quantitation shows the escape latency during acquisition trials. WT: n = 10, 5×FAD + Vehicle: n = 10, 5×FAD + Eda: n = 10, 5×FAD + Y-2: n = 10. two-way ANOVA with Fisher’s LSD post hoc analysis; ^aaa^*P*<0.001, 5×FAD + Vehicle vs. WT + Vehicle group; ^bbb^*P*<0.001, 5×FAD + Eda vs. 5×FAD Vehicle group. (D and E) Quantitation shows the swimming speed (**D**) and the percentage of time in the platform located quadrant (**E**) in the probe trial. WT + Vehicle: n = 10, 5×FAD + Vehicle: n = 9, 5×FAD + Eda: n = 9, 5×FAD + Y-2: n = 10. one-way ANOVA with Fisher’s LSD post hoc analysis; *P < 0.05, **P < 0.01, ***P < 0.001. (**F**) Representative trajectories show the visit frequency of mice in Y-maze. Arrow, the novel arm. (G-I) Quantification shows the speed (**G**), number of total entries (**H**) and percentage of novel arm entries (**I**). WT + Vehicle: n = 10, 5×FAD + Vehicle: n = 10, 5×FAD + Eda: n = 10, 5×FAD + Y-2: n = 10. one-way ANOVA with Fisher’s LSD post hoc analysis; *P < 0.05, ***P < 0.001. n represents individual animals. All data are represented as mean ± SD.

Due to the considerable difficulty of sublingual administration in mice, intraperitoneal (*i.p.*) injection was used in this study to evaluate the drug efficacy of Y-2 in the 5×FAD transgenic mice. As a drug in clinical trials, Y-2 concentration used in mice were calculated based on the FDA’s equation for extrapolation between human and mouse. Y-2 single sublingual administration (containing 30 mg of Eda and 6 mg of (+)-borneol) or a single *i.p.* injection of 7.5 mg/kg Y-2 (containing 6 mg/kg Eda and 1.5 mg/kg (+)-Borneol) displayed similar concentration-time curves (AUC_0 − inf_) in plasma (Eda: AUC_0 − inf_, 5290 vs. 5460 h ng/mL; borneol: AUC_0 − inf_, 30.8 vs. 30.2 h ng/mL) [30]. We then designed a workflow, and a schematic showed the experimental design (Fig. 1A). Previous findings have shown that female 5×FAD transgenic mice have more serious pathology [32] and heightened inflammation versus males [33]. Since our goal was to examine a later stage of pathology, we selected female 5×FAD in this study. Also, using one sex reduced variation in the cohort allowing the study to have sufficient power. Mice at 8 weeks old were separated into 4 indicated groups and each group of mice were treated by *i.p.* injection once daily with Vehicle, Eda (6 mg/kg), or Y-2 (7.5 mg/kg), respectively (Fig. 1A). After 16-weeks treatment for these mice, Morris water maze (MWM) and Y-maze were used to assess the cognitive function. The following procedure for histopathological study was depicted in Fig. 1A.

MWM assays demonstrated the cognitive improvement in Y-2-treated group, indicated by the decreased time in the escape latency during 5 days of acquisition trials (Fig. 1C), and more time spent in the target quadrant (Fig. 1B, E) in the probe trial with no speed differences (Fig. 1D) (One mouse in the 5×FAD vehicle group and one in the Eda-treated group barely swam in the probe trail, we thus excluded their MWMs data). Similarly, Y-2 treatment in the 5×FAD also showed an improvement in spatial working memory in Y-maze tests, compared to those treated with vehicle, indicating by the increased ratio of entering the novel arm during retrieval trials (Fig. 1F, I), with no differences observed in motor ability (Fig. 1G, H). In addition, the average levels of key behavioral indicators were upregulated (∼ 13.4% in MWM and ∼ 7.1% in Y-maze) by Y-2 compared to Eda alone (Fig. 1E, I). These data thus suggest that Y-2 is more effective than Eda alone on ameliorating cognitive defects of the 5×FAD mice.

## Y-2 mitigates Aβ-associated pathologies in the 5×FAD mice

**Fig. 2:**
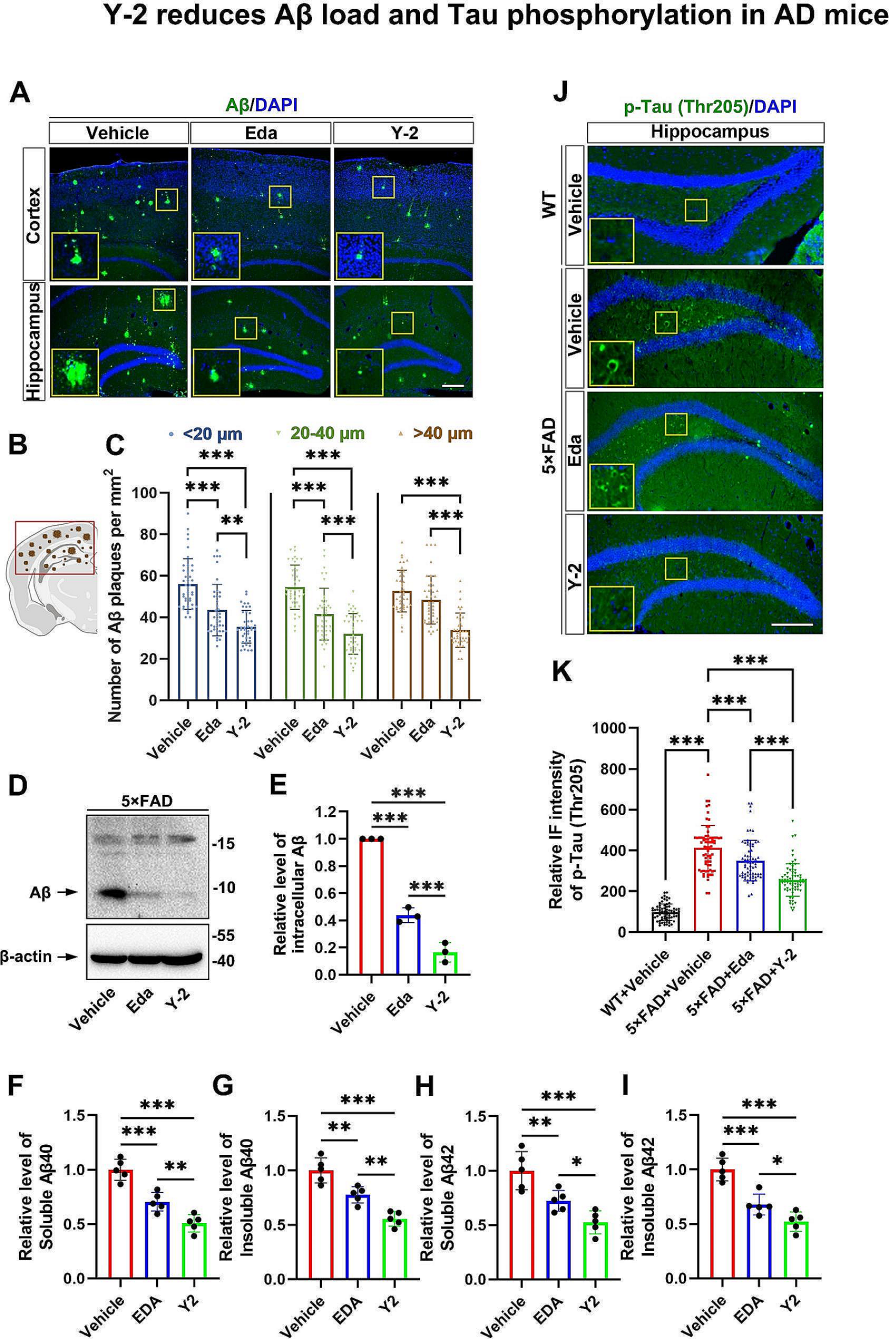
Y-2 reduces Aβ load and Tau phosphorylation in AD mice. (**A**) IHC staining of Aβ (green) in cortex and hippocampus of the 5×FAD mice. An anti- Aβ antibody (CST, #2454S) was used. Nuclei counter stained with DAPI (blue). (**B**) Graphical representation of coverage areas for Aβ plaque measurements in the brain of 5×FAD mice. (**C**) Quantification shows the average number of Aβ plaques with different sizes (diameter < 20 μm, 20–40 μm and > 40 μm) per mm^2^, n = 40. n represents the number of randomly selected slices from 5 mice in each group. one-way ANOVA with Fisher’s LSD post hoc analysis; *P < 0.05, **P < 0.01, ***P < 0.001. (**D**) Immunoblotting shows the levels of intracellular Aβ in brain lysate from randomly selected 3 mice. β-actin as a loading control. (**E**) Quantification of the blots in (**D**), n = 3. n represents the number of independent experiments. one-way ANOVA with Fisher’s LSD post hoc analysis; ***P < 0.001. The protein level in 5×FAD + Vehcile group was normalized to 1. (**F-I**) Relative levels of soluble and insoluble Aβ40 (**F** and **G**) and Aβ42 (**H** and **I**) in brain homogenates from randomly selected 5 mice. one-way ANOVA with Fisher’s LSD post hoc analysis; **P < 0.01; ***P < 0.001. (**J**) IHC staining of p-Tau (Thr205) (green) in hippocampal hilus of mice. (**K**) Histogram shows the IF intensity of p-Tau in (**J**), n = 70. n represents the cell number in hippocampal hilus on 15 slices from 5 mice in each group. one-way ANOVA with Fisher’s LSD post hoc analysis; **P < 0.01; ***P < 0.001. All quantification data are represented as mean ± SD. Scale bar, 100 μm

To evaluate whether Y-2 can alleviate Aβ pathology, a predominant cause for cognitive decline in the 5×FAD mice, we examined the Aβ-related histopathological features in cortex and hippocampus. As described before, 6-month-old 5×FAD mice have severe Aβ-associated pathological features such as Aβ deposition and gliosis in the brain [34]. After behavioral tests, 25-week-old mice were used to histological study (see Method). Immunohistochemical (IHC) analysis showed that after 4 months of treatment with Y-2, the average number of Aβ plaques of different sizes (diameter: <20 μm, 20–40 μm, and > 40 μm) all decreased in the cortical and hippocampal regions of the 5×FAD mice (Fig. 2A, C). The Y-2 group exhibited a reduction of ∼ 36.87% in < 20 μm diameter, ∼ 41.33% in 20–40 μm diameter, and ∼ 35.78% in > 40 μm diameter plaques compared to the vehicle group. Notably, Aβ plaques with medium and large sizes (diameter: 20–40 μm and > 40 μm) were significantly reduced by Y-2 compared to Eda treatment (Fig. 2A, C). In comparison to the Eda treatment, Y-2 reduced average numbers of plaques with diameters of < 20 μm, 20–40 μm, and > 40 μm by ∼ 18.61%, ∼ 23.03%, and ∼ 30.06%, respectively. Next, in order to evaluate the effect of Y-2 on intracellular Aβ levels, we examined the Aβ levels in the homogenate lysates of mouse brain by immunoblotting and found that Y-2 reduced intracellular Aβ in 5×FAD mice compared to vehicle (∼ 83.49%) (Fig. 2D, E). It is noteworthy that Y-2 also showed a strong reduction compared to Eda treatment (∼ 27.28%) (Fig. 2D, E). We next investigated the levels of Aβ40 and Aβ42 species in the diethylamine-extracted soluble and the formic acid-extracted insoluble fractions of brain homogenates, respectively. ELISA assays revealed that, in the Y-2 group compared to the vehicle, the levels of soluble Aβ40 and Aβ42 were decreased by ∼ 49.25% and ∼ 47.6%, and the insoluble Aβ40 and Aβ42 were decreased by ∼ 44.61% and ∼ 47.93% (Fig. 2F-I). Of note, when compared to Eda, Y-2 treatment led to a reduction in the levels of soluble Aβ40 and Aβ42 by ∼ 27.93% and ∼ 27.44%, and a decrease in insoluble Aβ40 and Aβ42 by ∼ 28.49% and ∼ 23.18%, (Fig. 2F-I).

Elevated Tau phosphorylation appears in the hippocampal hilus in both AD patients and the 5×FAD mice [35, 36]. Therefore, we used an antibody against phosphorylated-Tau (p-Tau) at Thr205 residue site, the most commonly analyzed site, to evaluate the p-Tau levels in the 5×FAD mice. Data showed that Y-2 treatment strongly reduced the levels of p-Tau (Thr205) in the hippocampal hilus and its efficacy is ∼ 27.09% higher than Eda alone (Fig. 2J, K).

**Fig. 3:**
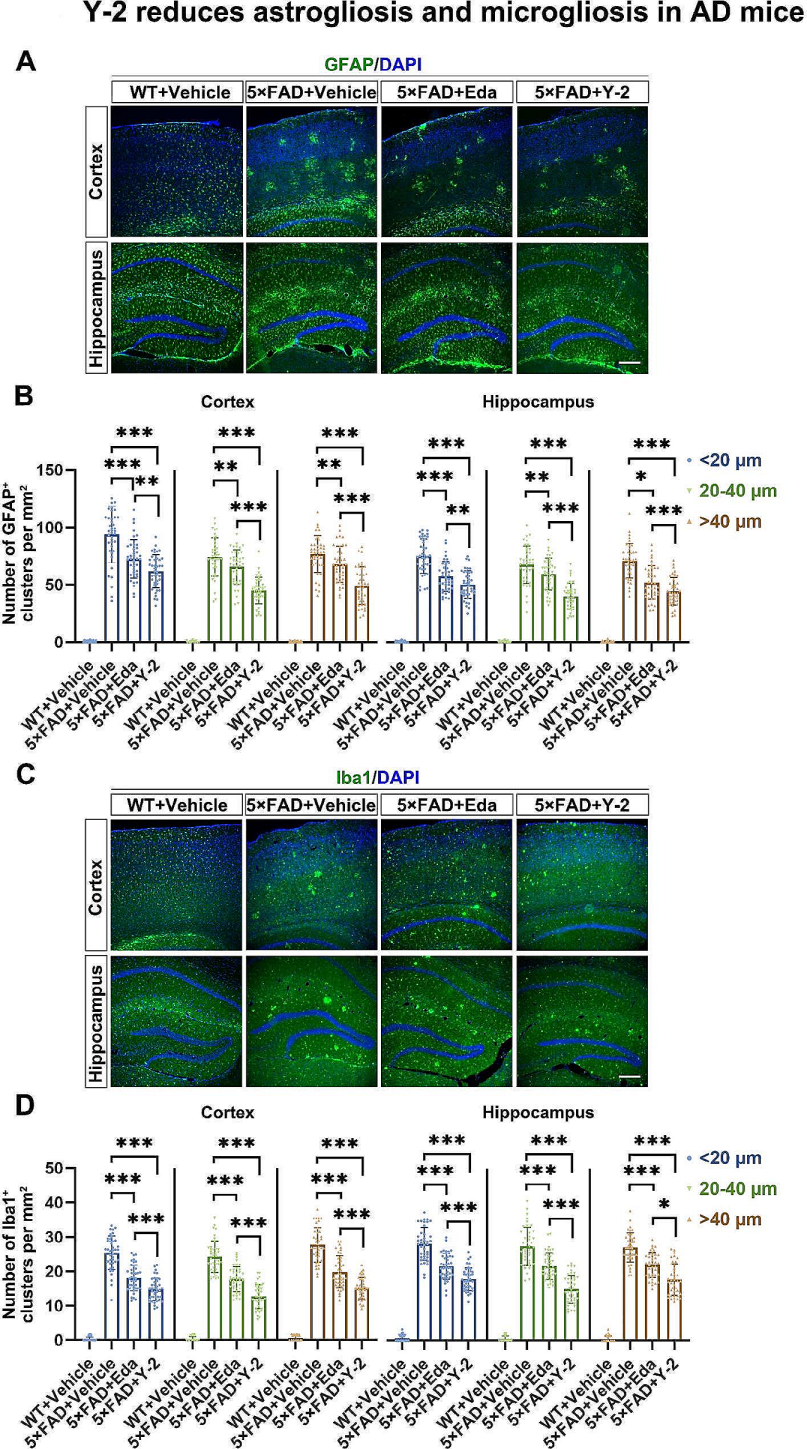
Y-2 reduces astrogliosis and microgliosis in AD mice. Images show the IHC staining of GFAP (green) (**A**) and Iba1 (green) (**C**) in cortex and hippocampus of the 5×FAD mice. Nuclei counterstained with DAPI (blue). Quantification shows the average number per mm^2^ of GFAP-positive astrocytic clusters with indicated sizes (diameter < 20 μm, 20–40 μm and > 40 μm) (**B**) and Iba1-positive microglial clusters (**D**). n = 40, n represents the number of randomly selected slices from 5 mice in each group. one-way ANOVA; *P < 0.05, **P < 0.01, ***P < 0.001. All data are represented as mean ± SD. Scale bar, 100 μm

Aβ deposits are usually accompanied by increased gliosis including astrogliosis and microgliosis in AD mice [34]. To confirm the efficacy of Y-2, we thus detected the levels of astrogliosis and microgliosis in the WT and 5×FAD mice treated with Vehicle, Eda or Y-2. IHC data showed that Y-2 decreased the numbers of astroglial and microglial clusters surrounding the plaques with each size (Fig. 3). Compared to Eda, Y-2 exhibited stronger inhibition on astroglial clusters by ∼ 15.08%, ∼ 31.45%, and ∼ 27.34% in cortex and ∼ 12.76%, ∼ 32.76%, and ∼ 14.61% in hippocampus with each size (Fig. 3B). Similarly, Y-2 showed stronger reduction on microglial clusters, with reductions of ∼ 18.6%, ∼ 28.48%, and ∼ 23.89% in cortex and ∼ 17.29%, ∼ 30.98%, and ∼ 19.24% in hippocampus with each different size (Fig. 3D).

We further investigated the role of Y-2 in the production and clearance of Aβ. We constructed a stable SH-SY5Y cell line overexpressing the Swedish mutant APP695. ELISA data showed that both Eda and Y-2 inhibited the levels of intracellular and extracellular Aβ40 and Aβ42 in APP695 SH-SY5Y cells, but with no significant difference of potency between the two drugs (Additional file 1: Fig. S2A-D). The more pronounced inhibitory effect of Y2 on Aβ deposition (Fig. 2A, C) raises the question of whether it is related to enhanced Aβ clearance. We thus evaluated the effect of Y-2 on the clearance of Aβ using a BV-2 microglial cell culture system. ELISA results showed that Y-2 could more effectively reduce the extracellular Aβ42 levels in culture media, possibly suggesting an enhanced Aβ clearance efficacy induced by Y-2 (Additional file 1: Fig. S2E).

Together, our data imply that Y-2 represses Aβ-associated pathologies in the 5×FAD mouse model of AD, with higher efficacy than Eda, by both inhibiting Aβ production and promoting Aβ clearance.

### Y-2 inhibits neuronal necroptosis and neuroinflammation in AD mice

**Fig. 4:**
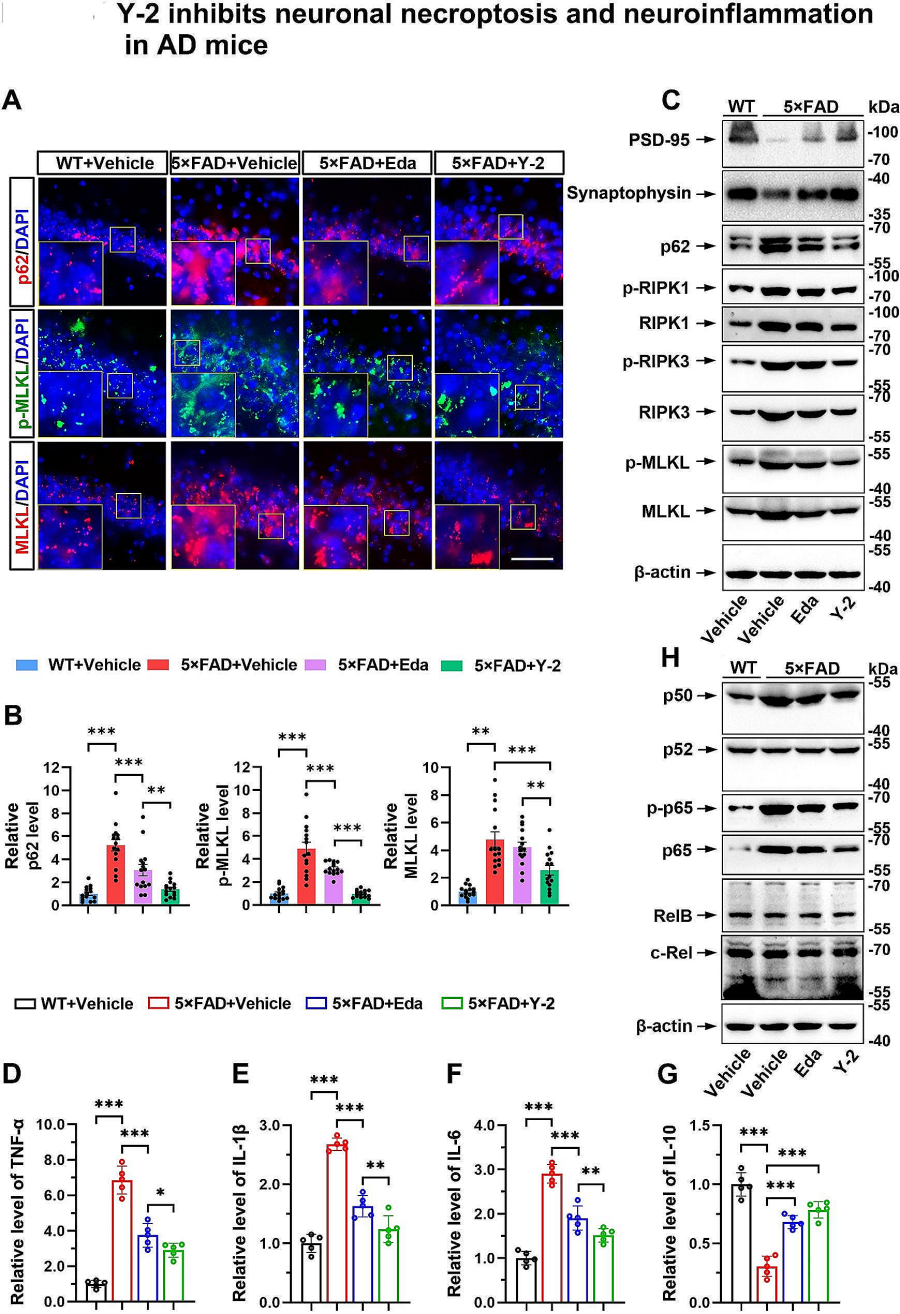
Y-2 inhibits neuronal necroptosis and neuroinflammation in AD mice. (**A**) IHC staining of p62 (red), p-MLKL (green) and MLKL (red) in hippocampcal CA1 of the 5×FAD mice. Nuclei counterstained with DAPI (blue). Scale bar, 100 μm. (**B**) Quantification of relative IF intensity of indicated proteins in (**A**), n = 15. n represents the number of randomly selected slices from 5 mice in each group. one-way ANOVA with Fisher’s LSD post hoc analysis; **P < 0.01, ***P < 0.001. The IF intensity in WT + Vehcile mice group were normalized to 1. (**C** and **H**) Immunoblotting shows the levels of indicated proteins in the brains. β-actin as a loading control. (**D**, **E**, **F** and G) ELISA shows the cerebral levels of TNF-α (**D**), IL-1β (**E**), IL-6 (**F**) and IL-10 (**G**), n = 5. n represents individual animals. one-way ANOVA with Fisher’s LSD post hoc analysis; *P < 0.05, **P < 0.01, ***P < 0.001. The concentration of the indicated cytokines in WT + Vehcile group was normalized to 1. All data are represented as mean ± SD

Recent findings from our group and others have shown that the activation of neuronal necroptosis is a critical event in AD pathogenesis [12, 37]. We speculated that the neuroprotective function of Y-2 might partially be due to its inhibition of neuronal necroptosis. To test this notion, we examined the protein levels of necroptotic markers in the brain lysates from WT and the 5×FAD mice in response to Vehicle, Eda or Y-2 treatment. IHC, and immunoblotting showed that Y-2 inhibited the reduction of PSD-95 (a postsynaptic marker) and Synaptophysin (a presynaptic marker), and repressed the accumulation of p62 as well as the activation of RIPK1/RIPK3/MLKL cascade (Fig. 4A-C). In accordance with our data in Figs. 2 and 3, IHC, and immunoblotting showed a higher efficacy of Y-2 than Eda, especially on the levels of p62, p-RIPK1/3 and p-MLKL (Fig. 4A-C).

The hyperactivation of the pro-inflammatory factor TNF-α in AD is thought to be one of the triggers of neuronal necroptosis [12, 13]. To confirm the inhibitory function of Y-2 on neuronal necroptosis, we further used an ELISA kit to evaluate the levels of TNF-α in brain lysates. Compared to WT, 5×FAD mice had a higher level of TNF-α in brain. Strikingly, elevated TNF-α was sharply reduced by ∼ 39.2% upon Eda treatment, and by ∼ 53.6% upon Y-2 treatment (Fig. 4D). We further extended to check other critical pro- and anti-inflammatory factors such as IL-1β, IL-6 and IL-10, which were all implicated in the regulation of neuroinflammation [38]. Consistently, ELISA data showed that the levels of pro-inflammatory IL-1β and IL-6 were markedly lowered by Eda or Y-2, while anti-inflammatory IL-10 was upregulated in the same state (Fig. 4E-G). It is noteworthy that the levels of TNF-α, IL-1β, and IL-6 in the brains of mice treated with Y-2 were lower compared to those mice treated with Eda. Although no statistical significance was observed between Y-2 and Eda, the average levels of IL-10 were higher in Y-2-treated brains. We also analyzed the levels of NF-κB subunits, which were considered as transcription factors promoting the production of multiple neural inflammatory cytokines including TNF-α [39]. Indeed, p65 and p50, but not other three subunits p52, RelB and c-Rel, were elevated in the brains of 5×FAD mice (Fig. 4H). Remarkably, either Eda or Y-2 treatment decreased the levels of p65 and p50, as well as those of phospho-p65 (Ser635), another major indicator of NF-κB activation (Fig. 4H).

Thus, our data demonstrate that both Eda and Y-2 can inhibit neuronal necroptosis and neuroinflammation in AD mice. The strong efficacy of Y-2, may contribute by the additional anti-inflammatory capacity of (+)-Borneol [28]. Interestingly, Y-2-mediated inhibition of neuronal necroptosis and neuroinflammation is thought to be due to the downregulation of total protein levels in these two pathways [37, 40], suggesting a possible transcriptional or translational regulation of those genes by Y-2 treatment.

### Y-2 reduces Aβ-induced neuronal damage in cell culture

**Fig. 5:**
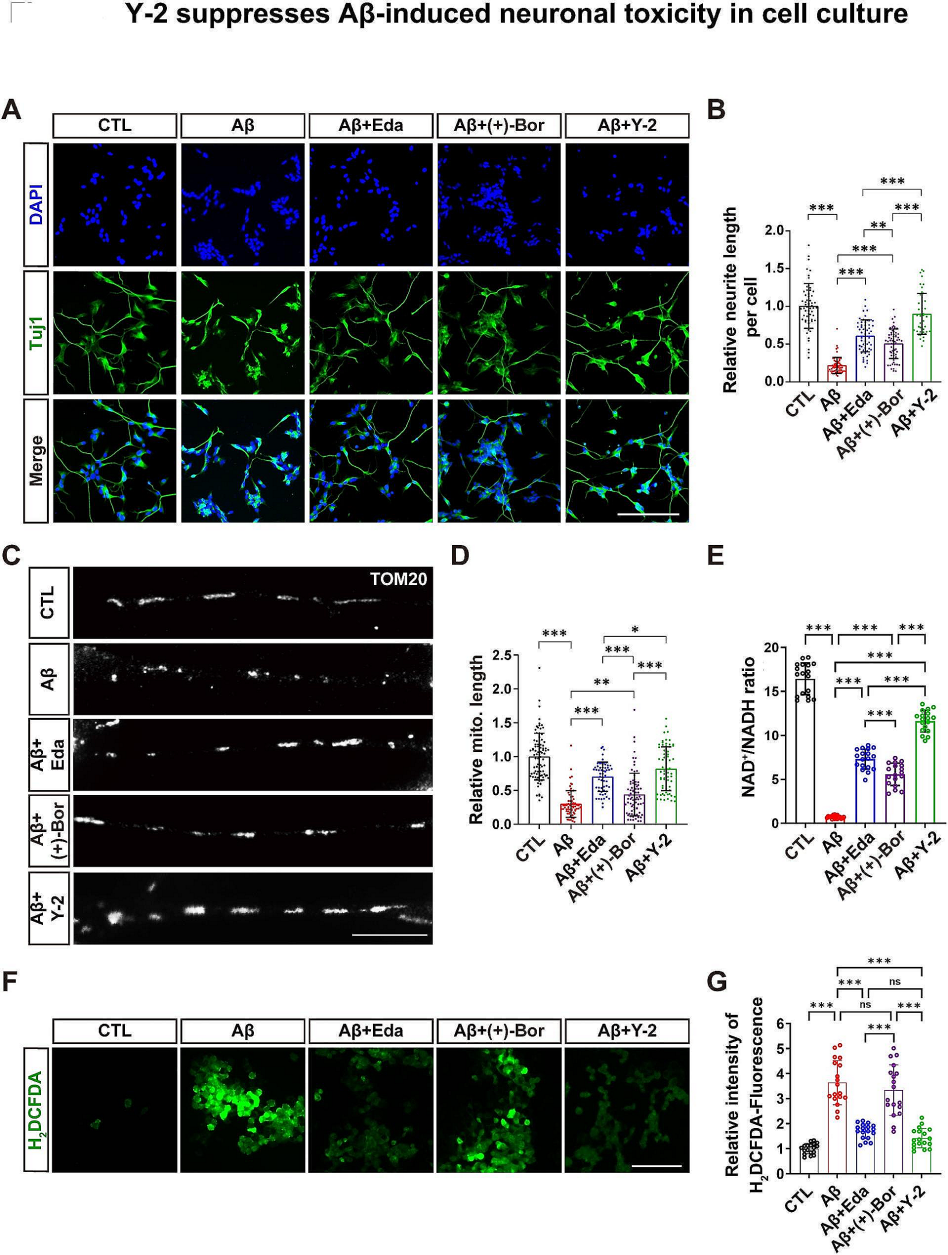
Y-2 suppresses Aβ-induced neuronal toxicity in cell culture. (**A**) IF of neuronal marker Tuj1 (green) in differentiated SH-SY5Y cells with indicated treatments (see Methods). Nuclei counterstained with DAPI (blue). Scale bar, 50 μm. (**B**) Quantification of neurite length in (A), n ≥ 40. n represents the number of random images from 3 independent experiments. one-way ANOVA with Fisher’s LSD post hoc analysis; **P < 0.01, ***P < 0.001. The neurite length per cell in CTL group was normalized to 1. (**C**) IF of TOM20 shows the mitochondrial segments in differentiated neurites of SH-SY5Y cells with indicated treatments. Scale bar, 10 μm. (**D**) The quantification shows the length of mitochondrial (mito.) segments in (**C**), n ≥ 20. n represents the number of randomly selected neurites from 3 independent experiments. one-way ANOVA with Fisher’s LSD post hoc analysis; *P < 0.05, **P < 0.01, ***P < 0.001. The mito. length in CTL group was normalized to 1. (**E**) Quantification of the NAD^+^/NADH ratio in SH-SY5Y cells with indicated treatments, n = 18. n represents the number of detected wells from 3 independent experiments. one-way ANOVA with Fisher’s LSD post hoc analysis; ***P < 0.001. (**F**) Images show the fluorescence intensity of H_2_DCFDA (Green, ROS indicator) in cells with indicated treatments. Scale bar, 50 μm. (**G**) Quantitation of relative H_2_DCFDA fluorescence intensity in (**F**), n = 18. n represents the number of detected wells from 3 independent experiments. one-way ANOVA with Fisher’s LSD post hoc analysis; **P < 0.01, ***P < 0.001, ns, not significant. The fluorescence intensity in CTL group was normalized to 1. All data are represented as mean ± SD.

In order to elucidate the neuroprotective mechanisms of Y-2, we used neuronal cell cultures and assessed the protective function of Eda and Y-2 against Aβ-induced neuronal damage. Consistent with the previous findings [17], Eda greatly reduced Aβ-triggered neurite injury in differentiated SH-SY5Y cells and primary neurons, indicated by the rescue of neurite length (Fig. 5A, B and Additional file 1: Fig. S3A, C). (+)-Borneol also showed a mild neurite protection against Aβ toxicity, while Y-2 had a better efficacy than Eda or (+)-Borneol (Fig. 5A, B and Additional file 1: Fig. S3A, C). Neurite injury is highly correlated to the impairment of mitochondria inside the neuronal processes. To assess whether Eda or Y-2 prevents mitochondrial damage stimulated by Aβ, we used immunofluorescence (IF) of TOM20 (a mitochondrial marker) to observe the morphology of mitochondria in SH-SY5Y cells and primary neurons. As expected, mitochondria in neurites were shortened and fragmented by Aβ stimulation. Again, Eda and Y-2 both repressed mitochondrial shortening and their fragmentation. Although (+)-Borneol alone also yielded partial protection against Aβ-induced mitochondrial damage, it had much less potency compared to Eda or Y-2 (Fig. 5C, D and Additional file 1: Fig. S3B, D). Next, we tested whether Y-2 exerted protective effects against NAD^+^ depletion and oxidative damage, two major pathological features induced by Aβ in AD models [41]. We determined the neuronal NAD^+^ levels by using a kit to measure the NAD^+^/NADH ratio. As expected, Aβ treatment sharply lowered the NAD^+^/NADH ratio in contrast to Vehicle (Fig. 5E). Indeed, Eda strongly increased the NAD^+^/NADH ratio by ∼ 7.7-fold (Fig. 5E). Of note, Y-2 had better efficacy than Eda, indicated by ∼ 11.6-fold upregulation of the NAD^+^/NADH ratio (Fig. 5E). Consistent with its mitochondrial protective function, (+)-Borneol alone also increased the NAD^+^/NADH ratio by ∼ 5.6-fold (Fig. 5E). Using a ROS fluorescent probe DHE, we further evaluated the ROS production in neuronal cells stimulated with Aβ oligomers. Data showed that intracellular ROS was elevated by ∼ 4.4-fold upon Aβ stimulation, while Eda strongly inhibited Aβ-induced ROS production by ∼ 67.2%. Y-2 showed a relative equal efficacy to Eda on ROS reduction, possibly due to no effect of (+)-Borneol (Fig. 5F, G).

Taken together, our data indicate that Y-2 serves as a strong neuroprotective agent against Aβ-triggered neuronal injury. In contrast to Eda, Y-2 has better efficacy in both mitochondrial and neurite protection.

### Y-2 activates anti-inflammatory pathways in astrocytes compared to Eda alone

**Fig. 6:**
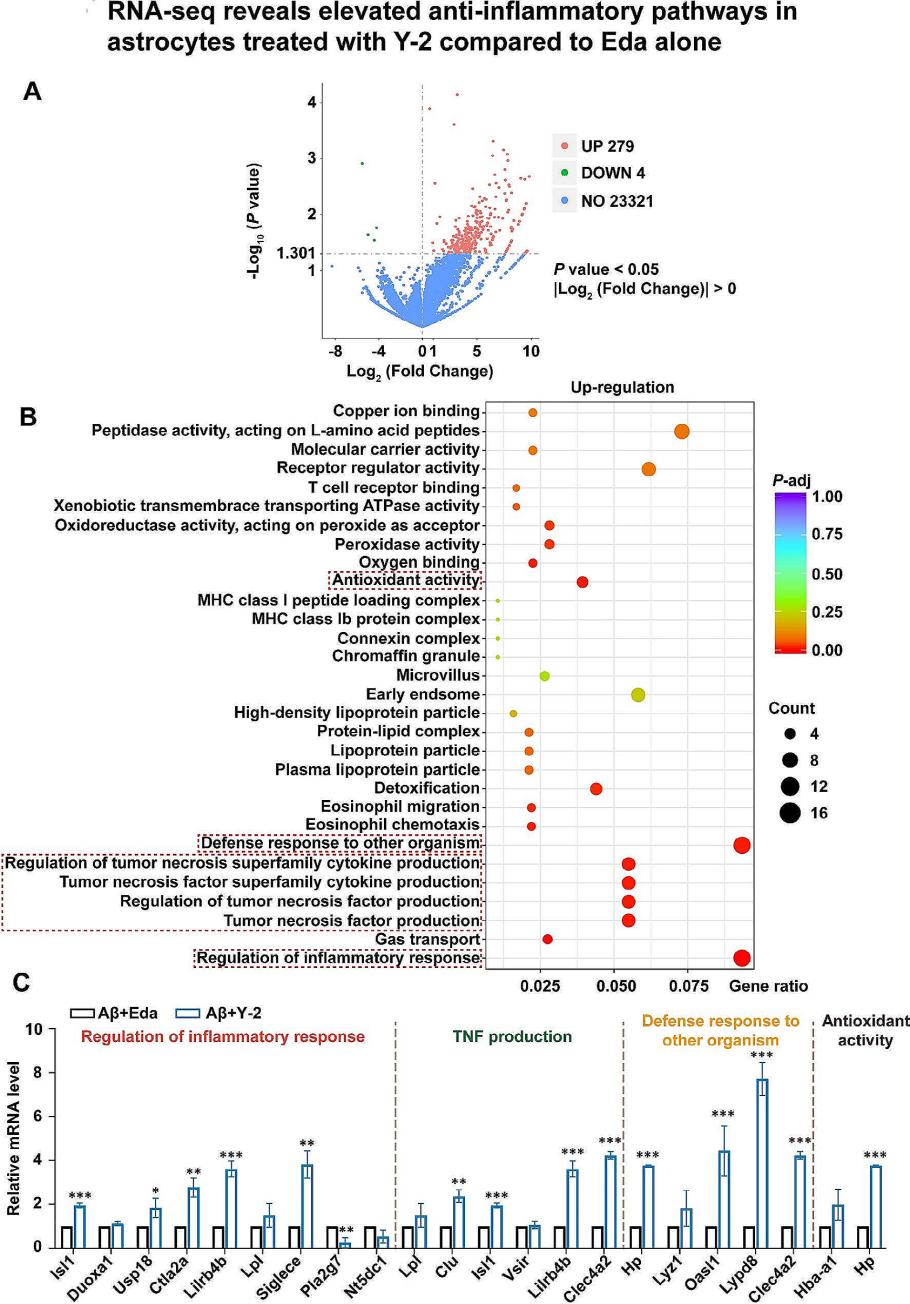
RNA-seq reveals elevated anti-inflammatory pathways in astrocytes treated with Y-2 compared to Eda alone. (**A**) Volcano plot of gene expression (Aβ + Y-2 versus Aβ + Eda; |log_2_ (fold change)| > 0; *P* value < 0.05). UP, up regulation; DOWN, down regulation; NO, no difference. (**B**) Gene Ontology (GO) enrichment analysis (Aβ + Y-2 versus Aβ + Eda). The vertical coordinates are the enriched pathways, and the horizontal coordinates are the gene ratio. The size of each point represents the number of upregulated genes in the pathway, and the color of the point represents the *P*-adjust. (**C**) mRNA levels of representative genes from indicated pathways are shown by real-time qPCR analyses, n = 3. n represents the number of independent experiments. *P < 0.05, **P < 0.01, ***P < 0.001; Student’s *t*-test. All data are represented as mean ± SD.

Our in vivo data in Fig. 4 have shown a novel function of Y-2 against neuronal necroptosis, which can be activated mainly by the most abundant astrocytes rather than microglia in several neurodegenerative diseases (NDs) [12, 42–44]. To further elucidate the molecular mechanism of Y-2 neuroprotection, we performed RNA-seq experiments to check the gene expression pattern in Aβ-stimulated astrocytes pre-treated with Eda, (+)-Borneol or Y-2. By comparison with Eda, Y-2 treatment led to a total 279 upregulated genes while only 4 downregulated genes (Fig. 6A). The Gene Ontology (GO) analysis showed that the differentially expressed genes (DEGs) were highly enriched in regulatory genes associated with immune regulation, tumor necrosis factor production, defense response to other organisms, and antioxidant activity (Fig. 6B). In accordance with these findings, (+)-Borneol alone, compared to Vehicle, also activates genes enriched in immune regulation (Additional file 1: Fig. S4), suggesting that anti-inflammatory efficacy of Y-2 may be derived from the (+)-Borneol compound.

To confirm the RNA-seq results, we carried out qPCR and examined genes involved in immune regulation (*Isl1*, *Duoxa1, Usp18*, *Ctla2a*, *Lilrb4b*, *Lpl*, *Siglece*, *Pla2g7*, and *Nt5dc1*), TNF production (*Lpl*, *Clu*, *Isl1*, *Vsir*, *Lilrb4b*, and *Clec4a2*), defense response to other organisms (*Hp*, *Lyz1*, *Oasl1*, *Lypd8*, and *Clec4a2*), and antioxidant activity (*Hba-a1* and *Hp*). Among these tested DGEs (Y-2 versus Eda), the expression of pro-inflammatory gene *Pla2g7* was remarkably reduced, while genes involved in anti-inflammatory pathways (*Isl1*, *Usp18*, *Ctla2a*, *Lilrb4b*, *Siglece*, *Clu*, *Clec4a2*, *Hp*, *Oasl1*, and *Lypd8*) were upregulated in C8-D1A astrocytic cells (Fig. 6C). Similarly, *Lypd8*, *Oasl1*, *Clec4a2*, *Hp*, *Isl1*, *Lilrb4b*, *Clu*, and *Siglece* were upregulated, and *Pla2g7* was reduced in primary astrocytes (Additional file 1: Fig. S5). These data thus show that Y-2 treatment, compared to Eda, has additional anti-inflammatory effects in astrocytes and may possibly inhibit astrocyte-mediated neuronal necroptosis.

### Y-2 inhibits astrocyte-mediated inflammation and represses neuronal necroptosis

**Fig. 7:**
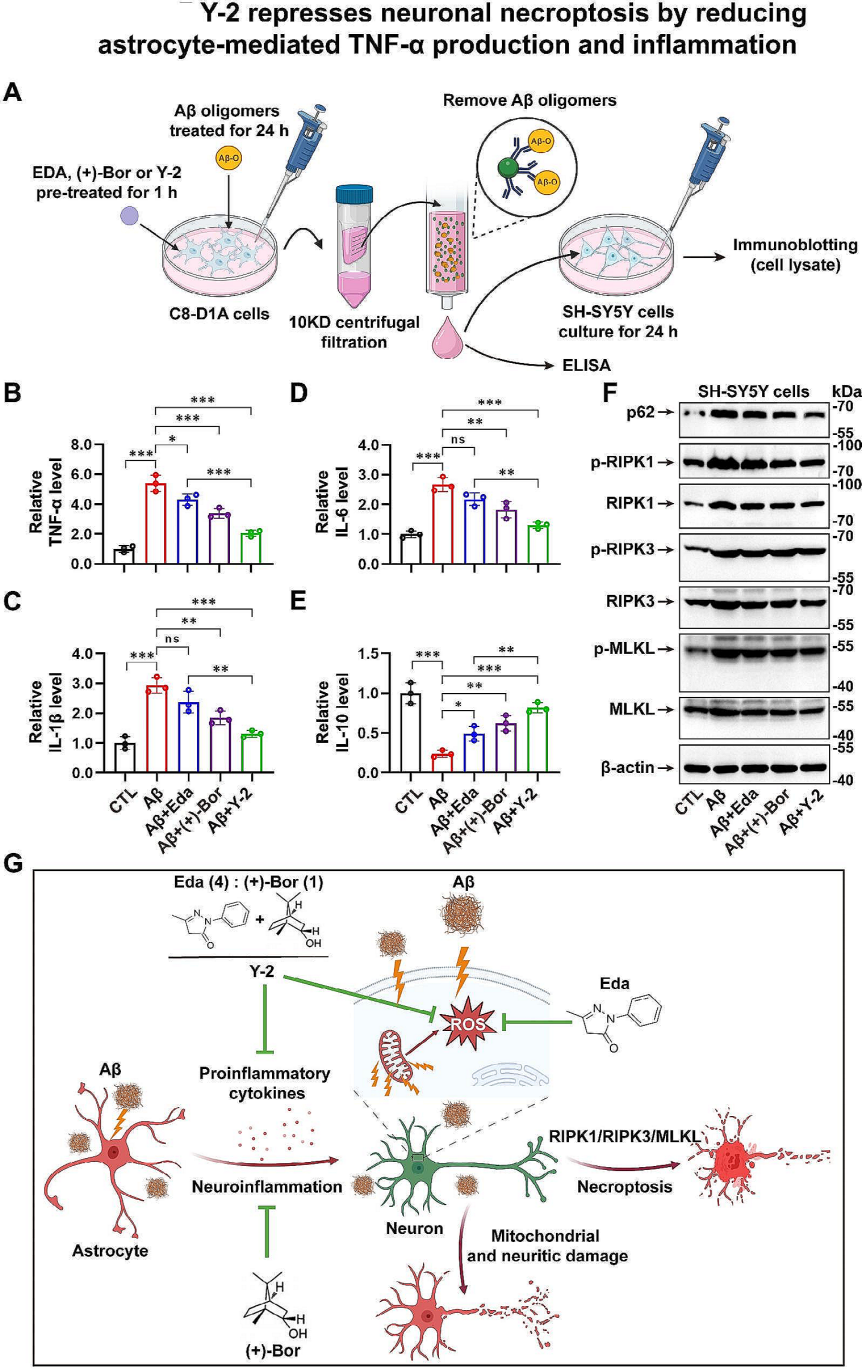
Y-2 represses neuronal necroptosis by reducing astrocyte-mediated TNF-α production and inflammation. (**A**) A schematic diagram shows the workflow of the astrocytic-neuronal culture procedure. ELISA data shows the levels of TNF-α (**B**), IL-1β (**C**), IL-6 (**D**) and IL-10 (**E**) in C8-D1A cell cultures, n = 3. n represents the number of independent experiments. one-way ANOVA with Fisher’s LSD post hoc analysis; *P < 0.05, **P < 0.01, ***P < 0.001, ns means not significant. All data are represented as mean ± SD. The concentration of the indicated cytokines in CTL group was normalized to 1. (**F**) Immunoblotting shows the protein levels of indicated necroptotic markers in SH-SY5Y cells treated with conditioned media from astrocytic C8-D1A cultures with indicated treatments. (**G**) A schematic summary of the present study

We next used a sequential astrocytic-neuronal cell culture system to address whether the inhibitory function of Y-2 on neuronal necroptosis and neuroinflammation is regulated by astrocytes, a major cell type responsible for TNF-α production and inflammation in brain [45]. As shown in a schematic diagram (Fig. 7A), we cultured the C8-D1A astrocytic cells and pre-treated them with Vehicle, Eda, (+)-Borneol or Y-2 for 1 h before Aβ stimulation. After 24 h treatment of Aβ, conditioned media (CM) was collected and drugs/Aβ oligomers inside were removed as described (Fig. 7A). The media was used for ELISA assays and data showed that Y-2 suppressed Aβ-induced TNF-α production by ∼ 62.9%, while Eda also reduced the TNF-α level by ∼ 20.4% (Fig. 7B). Consistent with its strong anti-inflammatory function, (+)-Borneol showed a ∼ 37% reduction of TNF-α (Fig. 7B). In addition, either Y-2 or (+)-Borneol markedly inhibited the production of other pro-inflammatory factors such as IL-1β and IL-6, while increased the levels of anti-inflammatory factor IL-10 (Fig. 7C-E). The CM was added into cultured SH-SY5Y cells and the cell activity and necrosis levels were measured using the CCK-8 assay and PI staining, respectively. Data showed that CM with Y-2 or (+)-Borneol alone pretreatment increased cell viability and prevented necrotic death (Additional file 1: Fig. S6). In contrast, CM with Eda alone pretreatment did not exert protective effect (Additional file 1: Fig. S6). Therefore, we investigated the levels of necroptotic markers in SH-SY5Y cells treated with CM from C8-D1A cell cultures (Fig. 7A). Immunoblotting showed that CM with Y-2 pretreatment reduced the levels of p62 and inhibited the activation of RIPK1/RIPK3/MLKL cascade (Fig. 7F). Interestingly, (+)-Borneol, as a powerful anti-inflammatory agent, indeed showed a mild inhibition on the activation of RIPK1, RIPK3 and MLKL (Fig. 7F).

Taken together, we conclude that Y-2 may inhibit the activation of neuronal necroptosis by repressing the astrocytic production of TNF-α and other pro-inflammatory factors.

## Discussion

In this study, we have demonstrated that the combination of Eda and (+)-Borneol (Y-2) provided stronger inhibition of cognitive deficits and AD pathologies in 5×FAD mice, suggesting that the combining antioxidant and anti-inflammatory drug synergizes to slow down AD progression, which is more efficacious than each alone in Aβ pathologies, neuronal necroptosis and NAD^+^ level, but not in anti-oxidant or anti-neuroinflammation. The animal behavioral data reveal that Y-2 treatment performed better in improving cognitive function of 5×FAD mice compared to Eda treatment (Fig. 1). Notably, Y-2 showed a stronger potency than Eda on reduction of AD pathologies (Figs. 2 and 3), in line with previous clinical findings indicating that Y-2 treatment had better outcomes than Eda alone in patients with AIS [31]. Given that Y-2 had a synergistic effect of anti-oxidant and anti-inflammatory, its better efficacy could possibly due to the additional anti-inflammatory effect of (+)-Borneol (Fig. 7).

Increasing evidence indeed suggests that AD pathogenesis is not restricted to neuronal compartments but is also closely related to neuroinflammatory mechanisms in the brain. Misfolded and aggregated proteins including Aβ oligomers/fibrils can activate recognition receptors on microglia and astrocytes and induce an innate immune response, featured by the release of inflammatory factors that contribute to AD progression [11]. Our data confirmed the activation of glial cells in AD, indicated by the astrogliosis and microgliosis surrounding Aβ plaques in the 5×FAD mice (Fig. 3). Strikingly, both Eda and Y-2 reduced the levels of gliosis (Fig. 3), inferring that glia-mediated inflammation was downregulated. Conformably, ELISA data from astrocytic culture and AD mice showed that Eda and Y-2 indeed lowered the levels of inflammatory factors such as IL-1β, IL-6 and TNF-α, but increased that of anti-inflammatory IL-10 (Figs. 4 and 7). Again, Y-2 exhibited a stronger anti-inflammatory efficacy than Eda alone (Fig. 7B-E) consistent with the strong anti-inflammatory function of (+)-Borneol [46].

The inflammatory TNF-α is also a crucial triggering factor responsible for neuronal necroptosis in AD [12]. We thus extended our study into neuronal necroptosis. In our sequential astrocytic-neuronal culture model, we found that Aβ oligomers induced NF-κB activation in astrocytes and increased ∼ 5-fold production of TNF-α, which further triggered the activation of necroptotic cascade in neuronal cells (Fig. 7B, F). In vivo data also confirmed the activation of NF-κB and neuronal necroptosis in AD mice (Fig. 4). More importantly, both Eda and Y-2 showed a good efficacy against NF-κB activation and neuronal necroptosis (Fig. 4). It is worth pointing out that Y-2 mainly reduces the total protein levels of NF-κB subunits as well as the key proteins in necroptotic pathway (Figs. 4 and 7). Whether and how Y-2 inhibits the transcription or translation of these genes, deserves further study in the future.

According to the survey report of “Alzheimer’s disease drug development pipeline: 2022”, 31 drugs for AD treatment are currently in phase 3 clinical trials [47], including 3 antioxidants Hydralazine, Icosapent ethyl (the purified product of eicosapentaenoic acid (EPA)) and Omega-3 (a complex of docosahexaenoic acid (DHA) and EPA). Hydralazine exerts antioxidant effects by activating nuclear factor E2-related factor 2 (Nrf2) [48]. Both EPA and DHA, as Omega-3 polyunsaturated fatty acids (PUFAs), have been reported to have neuroprotective function through their antioxidant effects, and supplementation of DHA and/or EPA can alleviate AD symptoms [49, 50]. Interestingly, most of the studies also have reported the therapeutic effects of Omega-3 PUFAs against AD involve the anti-inflammatory effect [49, 50]. As far, there are currently 17 anti-inflammatory drugs in Phase 2 clinical trials (20.73% of total Phase 2 clinical candidates), including 9 biological reagents directly modulating immunity and 8 anti-inflammatory compounds targeting different immune proteins. Based on these new studies, strategies of anti-oxidation and anti-neuroinflammation are both important directions of current drug development against AD. Y-2, as a combination of Eda and (+)-Borneol, not only exerts superior antioxidant effect of Eda (Fig. 5F, G), but also inhibits neuronal necroptosis and neuroinflammation through the anti-inflammatory effect of (+)-Borneol (Figs. 4 and 7). Although both Eda and Y-2 treatments have been clinically reported with adverse effects including bruising, problems walking, and headaches; no significant difference in severe adverse events was found between Eda and Y-2 treatments in AIS patients [51]. Thus, we consider that Y-2 is worthy of further clinical investigation for AD treatment in the future.

## Conclusions

In summary, we demonstrate that Y-2 attenuates cognitive deficits and AD-related pathologies in 5×FAD mice. In addition, we identify a mechanism by which Y-2 exerts a more potent effect than Eda alone, specifically in suppressing astrocyte-mediated neuroinflammation and subsequent neuronal necroptosis. Although our findings lead us to consider Y-2 as a strong drug candidate for AD therapy, this study still has several limitations. For instance, we need to investigate: (1) Y-2 efficacy in male 5×FAD mice, (2) Y-2 efficacy in other AD animal models, e.g. a Tau model, (3) Y-2 pharmacokinetic studies in AD animal models, (4) Y-2 efficacy in aged mice (with treatment started after amyloid pathology), (5) Y-2 may affect microglia-mediated inflammation and Aβ clearance. However, our findings support the notion that Y-2 treatment has promising efficacy against AD progression, and may also have general neuroprotective function in other NDs.

## Materials and methods

### Drugs and mice

Edaravone injection (10 mg/5 mL) and Y-2 injection containing Edaravone (10 mg/5 mL) and (+)-Borneol (2.5 mg/5 mL) were provided by NeuroDawn Pharmaceutical Co., Ltd. Eda or Y-2 injections were diluted in saline and injected by *i.p.* with 6 mg/kg (Eda), or 7.5 mg/kg (Y-2), once daily. Female C57BL/6 wild-type (WT) and 5×FAD (JAX, Stock # 034848) mice were used in this study. 8-week-old 5×FAD mice were randomly divided into 3 groups (n = 10), injected with saline (Vehicle), Eda or Y-2 for further use. WT mice were injected with saline as a control group. All mice were maintained in a temperature (26–28 °C) and humidity-controlled environment with a 12-h light-dark cycle and free access to food and water unless otherwise indicated. All experiments were conducted in accordance with the regulations for the Administration of Affairs Concerning Experimental Animals and approved by the Laboratory Animal Care Committee at the China Pharmaceutical University (Permit number SYXK-2021-0011).

### Morris water maze (MWM) and Y-maze

Morris water maze (MWM) assays were performed using the same protocol we described previously [12]. In brief, mice were trained to locate a covert escape platform in a circular pool with a depth of 60 cm, a diameter of 130 cm, and a constant temperature of 22 °C. The platform, which had a diameter of 10 cm, was in the center of the southwest quadrant, 1 cm below the water’s surface. One day prior to the training, all mice were tested by a visible platform to evaluate the suitability of their motor function. The training spanned five days, with each mouse participating in four sessions daily. Mice were released from various entry points (north, east, south-east, and north-west) towards the pool wall, with the order changing each day. Swimming behavior was recorded using video tracking for a maximum of 60 s or until the mouse reached the concealed escape platform and remained there for at least 2 s. If a mouse took longer than 60 s to find the platform, it was guided to it and allowed to stay for at least 10 s. Following the acquisition trials, the escape platform was removed, and the mice were released facing the pool wall at the north-east entry point. During the probe trials, swimming behavior was video-tracked automatically for 60 s.

Y-maze experiments were conducted as previously conducted [52]. Briefly, 3 identical arms of the maze were randomly designated as the starting arm, the novel arm, and the other arm. The training trial was 15 min and mice were only allowed to explore 2 arms by blocking the novel one. For retrieval trial, mice were placed back in the same starting arm and allowed exploration for 5 min with free access to all 3 arms. The whole process was captured by a ceiling-mounted CCD camera and the data were analyzed by the software following the manufacturer’s protocol.

### Animal tissue collection and grouping

After the behavioral tests, all mice were euthanized with an overdose of anesthetics and then perfused transcardially with saline. Subsequently, all the total 10 brain tissues from each group were collected and separated into 2 hemispheres for following experiments. Within each group, we randomly selected the 5 hemispheres from different mice for IHC (n = 5) and another 3 hemispheres from different mice for immunoblotting (n = 3). Next, we randomly selected 5 hemispheres from different mice for ELISA (n = 5).

### Immunoblotting, immunohistochemistry (IHC) and immunofluorescence (IF)

Immunoblotting was performed according to our standard protocol [53]. For the protein extraction, the perfused hemispheres were homogenized and lysed using a kit (Genuin Biotech) following the manufacturer’s protocol. For IF of cultured cells, cells on coverslips were fixed in 4% paraformaldehyde (PFA) and followed a protocol reported in our previous paper [12]. For IHC of brain tissues, the perfused hemispheres were fixed in 4% PFA for 24 h, and dehydrated in a 30% PBS-buffered sucrose solution for around 24 h until tissue sinks. Next, the tissues were embedded in optimal cutting temperature compound (OCT) for cryostat sectioning. 30 μm cryosections were cut using a Leica CM1950 microtome. IHC were conducted using our standard protocol as described [12]. The primary antibodies used in this study were listed as below: anti-MLKL (sc-165,025, RRID: AB_10839183), anti-p50 (sc-1190, RRID: AB_632033), anti-p52 (sc-298, RRID: AB_2151277) and anti-c-Rel (sc-70-G, RRID: AB_632042) were purchased from Santa Cruz; anti-β-Amyloid (2454, RRID: AB_2056585), anti-p-Tau (Thr205) (49,561, RRID: AB_2799361), anti-PSD-95 (3450, RRID: AB_2292883), anti-Synaptophysin (36,406, RRID: AB_2799098), anti-p65 (8242, RRID: AB_10859369), anti-p-p65 (3033, RRID: AB_331284), anti-p62 (39,749, RRID: AB_2799160), anti-RIP (3493, RRID: AB_2305314), anti-p-RIPK1 (S166) (65,746, RRID: AB_2799693) and anti-Tuj1 (5568, RRID: AB_10694505) were purchased from Cell Signaling Technology; anti-GFAP (ab7260, RRID: AB_305808), anti-RelB (ab71543, RRID: AB_1861760), anti-p-MLKL (S345) (ab196436, RRID: AB_2687465), anti-p-MLKL (S358) (ab187091, RRID: AB_2619685), anti-p-RIPK3 (T231/S232) (ab205421, RRID: AB_2868433) and anti-p-RIPK3 (S227) (ab209384, RRID: AB_2714035) were purchased from Abcam; anti-Iba1 (GTX100042S, RRID: AB_10723913) was purchased from GeneTex; anti-RIPK3 (A5431, RRID: AB_2766237) and anti-Actin (AC004, RRID: AB_2737399) were purchased from Abclonal; anti-TOM20 (11,802, RRID: AB_2919606) was purchased from Proteintech. All the secondary antibodies used in this study were purchased from Thermo.

### Aβ pathology assessment

We measured the number of Aβ plaques of various diameters in the cortex and hippocampus (as shown in Fig. 2B), categorized into three groups: <20 μm, 20–40 μm, and > 40 μm. We then calculated the number of Aβ plaques of different diameters per mm^2^ in the cortexes and hippocampi. For p-Tau detection, we evaluated the fluorescence intensity of p-Tau in a fixed-size random area within the hippocampal hilus of mice. For gliosis measurement, we separately quantified the number of astroglial and microglial clusters of various diameters (divided into < 20 μm, 20–40 μm, and > 40 μm groups) in the cortexes and hippocampi, and calculated the number of gliosis per mm^2^.

### Cell culture

SH-SY5Y, BV-2 and C8-D1A cells (ATCC) were cultured in DMEM with 10% FBS. Primary neuronal cultures were performed as described [12]. In brief, cortical neurons were isolated from brains of P0 mice and cultured in Neurobasal (Gibco) medium supplemented with B-27 (Gibco) on Poly-D-Lysine (PDL) (Sigma Aldrich) coated 6-well plates. The mouse primary astrocytes were isolated from the brains of P0 mice. In brief, the brain tissues were digested and cell pellets were resuspended in DMEM with 10% FBS. Cells are cultured in PDL coated T-75 flasks to allow astrocytes to adhere and proliferate, while non-adherent cells were removed through medium changes. After reaching confluence, astrocytes were purified using shaking detachment methods, ensuring a high purity culture. The APP695-SH-SY5Y stable cell line was constructed by transducing SH-SY5Y cells with lentiviral particles expressing the Swedish mutant APP695. For Aβ production study, APP695-SH-SY5Y cells were incubated with or without Eda (2.4 µM) or Y-2 (3 µM) for 72 h followed by ELISA tests for Aβ40 and Aβ42. For Aβ clearance study, BV-2 cells were pre-treated with Eda (2.4 µM) or Y-2 (3 µM) for 1 h followed by 5 µM soluble Aβ42 treatment of 12 h. Subsequently, the residual Aβ42 levels in the culture media were detected by ELISA. For neuronal differentiation, SH-SY5Y cells were seeded on PDL coated 6-well plates containing Neurobasal medium plus with B-27 and trans-Retinoic acid (Abcam), then cultured for 5 days until use. For Aβ toxicity study, cells were incubated with 5 µM Aβ oligomers for 72 h with or without Eda (2.4 µM), (+)-Borneol (0.6 µM), or Y-2 (3 µM) pretreatment for 1 h. For the sequential astrocytic-neuronal culture, C8-D1A cells were incubated with 5 µM Aβ oligomers for 24 h with pretreatment of Vehicle, Eda (2.4 µM), (+)-Borneol (0.6 µM), or Y-2 (3 µM) for 1 h. The conditioned media was filtered by a 10 KD centrifugal filtration column (Millipore) to remove drugs. After that, media was further filtered by an affinity column containing anti-Aβ oligomers beads (Millipore) to remove the Aβ oligomers. The purified media was used for ELISA assays, or added into SH-SY5Y cells as culture media for another 24 h culture until cell viability assay, PI staining or cell lysate extraction and immunoblotting experiments.

### Preparation of Aβ oligomers

Aβ42 peptides were purchased from GenScript. The oligomerization of Aβ42 was conducted as described [54]. In brief, 1 mM Aβ42 peptides were dissolved in hexafluoroisopropanol (HFIP) to remove pre-existing aggregates and β-sheet secondary structure. After 30 min incubation at RT, aliquots of Aβ42 were moved to a SpeedVac and dried for 1 h to fully evaporate the HFIP, then stored at -80 °C. Prior to use, Aβ42 films were dissolved at 5 mM in DMSO, vortexed for 30 s, and sonicated in a water bath for 10 min, then incubated at 4 °C for 24 h for oligomerization. The formation of Aβ oligomers was detected using immunoblotting (Additional file 1: Fig. S1).

### Morphological analysis of neurites and mitochondria

For neurite analysis, brightfield or fluorescent images were acquired using an Olympus IX73 microscope. The total length of neurites and the number of cells were measured using ImageJ software. Subsequently, the average total length of neurites per cell was calculated. To ensure statistical significance, at least 20 random images from 3 different experiments were utilized for quantification. To investigate mitochondrial morphology, images were captured using a 60× objective (oil, NA1.4). The length of mitochondria in neurites were assessed and computed using ImageJ software. A minimum of 5 mitochondrial segments were analyzed in each neurite, with a total of more than 20 neurites considered.

### Detection of ROS and NAD^+^/NADH

The cellular ROS assays were performed using an oxidant-sensitive probe H_2_DCFDA (MCE). Briefly, cells were washed and incubated with PBS containing 10 µM H_2_DCFDA for 20 min, then quickly washed three times with 37 °C PBS for capturing images under a fluorescence microscope (Olympus, IX73). The NAD^+^/NADH ratio was determined using an NAD^+^/NADH Assay Kit (Beyotime). In brief, cells were lysed with extraction buffer and the supernatants were collected by centrifugation. The lysates were added into 96-well plates for data collection using a microplate spectrophotometer (Molecular Devices).

### Soluble and insoluble Aβ extraction

Soluble and insoluble Aβ were extracted from the mouse brain homogenates using diethylamine (DEA) and formic acid (FA), respectively. In brief, the brains were homogenized in ice-cold Tris-buffered saline (TBS) containing a protease inhibitor cocktail (Sigma) and ethylenediaminetetraacetic acid (EDTA). The soluble Aβ was extracted using cold 0.4% DEA in 100 mM NaCl, centrifuged at 135,000 × g for 1 h at 4 °C, and then neutralized by adding 0.5 M Tris-HCl, pH 6.8. The insoluble Aβ was extracted from remaining pellet using 70% cold FA, sonicated for 1 min, centrifuged as above, and neutralized in TBS containing 0.5 M Na_2_PO_4_ and 0.05% NaN_3_.

### ELISA

The levels of Aβ40 and Aβ42 were detected using Aβ40 and Aβ42 ELISA Kits (Shanghai Yaji biological technology). The intracellular and extracellular Aβ were collected from cell lysates or the culture medium, respectively. Subsequently, the samples were added into 96-well plates pre-coated with the Aβ40 or Aβ42 antibodies, following the manufacturer’s protocols. The levels of inflammatory factors were examined using ELISA Assay Kits (Jiangsu Meibiao Biotechnology) to detect mouse TNF-α, IL-1β, IL-6 and IL-10, respectively. In brief, the fresh mouse brain homogenates or the conditioned media of cultured C8-D1A cells were added into 96-well plates pre-coated with the capture antibodies, according to the manufacturer’s instruction. After incubation and elution, the 96-well plates were read at a wavelength of 450 nm by a microplate spectrophotometer (Molecular Devices).

### RNA-Seq and RT-qPCR

Total RNA from C8-D1A cells was extracted using RNA-easy™ Isolation Reagent (Vazyme), according to the operation manual. RNA library was prepared for transcriptome sequencing, which was conducted by Novogene Co., LTD (Beijing, China). Differentially expressed genes were counted with adjusted *P* value < 0.05 and |log_2_(Fold Change)| > 0. According to the manufacturer’s instruction, 1 µg total RNA was converted to complementary DNA (cDNA) using a HiScript 1st Strand cDNA Synthesis Kit (Vazyme). The real-time PCR (qPCR) was performed using our standard protocol. Primer sequences were listed in Supplementary Table 1.

### Cell viability assay and propidium iodide (PI) staining

For cell viability assay, SH-SY5Y cells were seeded in 96-well plates before CM treatment. The cells were treated with reagents in Enhanced Cell Counting Kit-8 (CCK-8) (Beyotime), and the procedure was conducted according to the manufacturer’s manual. For PI staining, SH-SY5Y cells were seeded in 6-well plates before CM treatment. The cells were then incubated with 2 µg/ml PI (Sigma Aldrich) for 10 min. After that, cells were washed with 37 °C PBS 3 times, followed by image capture using Olympus IX73 microscope. The percentage of necrotic cells was calculated by dividing the number of PI-positive cells by the total number of cells per well.

### Data and statistical analyses

All data were presented as mean ± SD. Data normality was examined by the Shapiro–Wilk test. Student’s *t*-test was used to identify statistically significant differences between two groups or one-way or two-way (Fig. 1C) analysis of variance (ANOVA) with Fisher’s LSD post hoc multiple comparison test for three or more groups. GraphPad Prism 8.0 was used for the statistical analysis. *P* values < 0.05 was considered statistically significant.

### Electronic supplementary material

Below is the link to the electronic supplementary material.

Supplementary Material 1

## Data Availability

All data are available from the authors upon request.

## Abbreviations

Aβ: amyloid-beta
ACSF: artificial cerebrospinal fluid
AD: Alzheimer’s disease
AIS: acute ischemic stroke
ALS: amyotrophic lateral sclerosis
(+)-Bor: (+)-Borneol
CM: conditioned media
Eda: Edaravone
HFIP: hexafluoroisopropanol
IF: immunofluorescence
IHC: immunohistochemistry
DEA: diethylamine
FA: formic acid
TBS: Tris-buffered saline
EDTA: ethylenediaminetetraacetic acid
MLKL: mixed lineage kinase domain-like
MWM: Morris water maze
NAD^+^: nicotinamide adenine dinucleotide
ND: neurodegenerative disease
RIPK1/3L: receptor-interacting serine/threonine-protein kinase 1 and 3
ROS: reactive oxygen species
WT: wild-type
Y-2: Edaravone Dexborneol

## References

[1] Hardy J, Selkoe DJ (2002). The amyloid hypothesis of Alzheimer’s disease: progress and problems on the road to therapeutics. Science.

[2] Selkoe DJ (2002). Deciphering the genesis and fate of amyloid beta-protein yields novel therapies for Alzheimer disease. J Clin Invest.

[3] Hou Y, Song H, Croteau DL, Akbari M, Bohr VA (2017). Genome instability in Alzheimer disease. Mech Ageing Dev.

[4] Jagust W (2018). Imaging the evolution and pathophysiology of Alzheimer disease. Nat Rev Neurosci.

[5] Hardy JA, Higgins GA (1992). Alzheimer’s disease: the amyloid cascade hypothesis. Science.

[6] Leng F, Edison P (2021). Neuroinflammation and microglial activation in Alzheimer disease: where do we go from here?. Nat Reviews Neurol.

[7] Kametani F, Hasegawa M (2018). Reconsideration of amyloid hypothesis and tau hypothesis in Alzheimer’s Disease. Front NeuroSci.

[8] Christen Y (2000). Oxidative stress and Alzheimer disease. Am J Clin Nutr.

[9] Hou Y, Lautrup S, Cordonnier S, Wang Y, Croteau DL, Zavala E, Zhang Y, Moritoh K, O’Connell JF, Baptiste BA (2018). NAD(+) supplementation normalizes key Alzheimer’s features and DNA damage responses in a new AD mouse model with introduced DNA repair deficiency. Proc Natl Acad Sci U S A.

[10] Wyss-Coray T (2006). Inflammation in Alzheimer disease: driving force, bystander or beneficial response?. Nat Med.

[11] Heneka MT, Carson MJ, El Khoury J, Landreth GE, Brosseron F, Feinstein DL, Jacobs AH, Wyss-Coray T, Vitorica J, Ransohoff RM (2015). Neuroinflammation in Alzheimer’s disease. Lancet Neurol.

[12] Xu C, Wu J, Wu Y, Ren Z, Yao Y, Chen G, Fang EF, Noh JH, Liu YU, Wei L (2021). TNF-alpha-dependent neuronal necroptosis regulated in Alzheimer’s disease by coordination of RIPK1-p62 complex with autophagic UVRAG. Theranostics.

[13] Jayaraman A, Htike TT, James R, Picon C, Reynolds R (2021). TNF-mediated neuroinflammation is linked to neuronal necroptosis in Alzheimer’s disease hippocampus. Acta Neuropathol Commun.

[14] De Felice FG, Velasco PT, Lambert MP, Viola K, Fernandez SJ, Ferreira ST, Klein WL (2007). Abeta oligomers induce neuronal oxidative stress through an N-methyl-D-aspartate receptor-dependent mechanism that is blocked by the Alzheimer drug memantine. J Biol Chem.

[15] Cheignon C, Tomas M, Bonnefont-Rousselot D, Faller P, Hureau C, Collin F (2018). Oxidative stress and the amyloid beta peptide in Alzheimer’s disease. Redox Biol.

[16] Dumont M, Beal MF (2011). Neuroprotective strategies involving ROS in Alzheimer disease. Free Radic Biol Med.

[17] Jiao SS, Yao XQ, Liu YH, Wang QH, Zeng F, Lu JJ, Liu J, Zhu C, Shen LL, Liu CH (2015). Edaravone alleviates Alzheimer’s disease-type pathologies and cognitive deficits. Proc Natl Acad Sci U S A.

[18] Hou Y, Wei Y, Lautrup S, Yang B, Wang Y, Cordonnier S, Mattson MP, Croteau DL, Bohr VA. NAD(+) supplementation reduces neuroinflammation and cell senescence in a transgenic mouse model of Alzheimer’s disease via cGAS-STING. Proc Natl Acad Sci U S A 2021, 118. 10.1073/pnas.2011226118 PMC8449423 34497121

[19] Fang EF, Lautrup S, Hou Y, Demarest TG, Croteau DL, Mattson MP, Bohr VA (2017). NAD(+) in aging: Molecular mechanisms and translational implications. Trends Mol Med.

[20] Kaltschmidt B, Uherek M, Volk B, Baeuerle PA, Kaltschmidt C (1997). Transcription factor NF-kappaB is activated in primary neurons by amyloid beta peptides and in neurons surrounding early plaques from patients with Alzheimer disease. Proc Natl Acad Sci U S A.

[21] Ferrer I, Marti E, Lopez E, Tortosa A (1998). NF-kB immunoreactivity is observed in association with beta A4 diffuse plaques in patients with Alzheimer’s disease. Neuropathol Appl Neurobiol.

[22] Seo EJ, Fischer N, Efferth T (2018). Phytochemicals as inhibitors of NF-kappaB for treatment of Alzheimer’s disease. Pharmacol Res.

[23] Writing G, Edaravone ALSSG (2017). Safety and efficacy of edaravone in well defined patients with amyotrophic lateral sclerosis: a randomised, double-blind, placebo-controlled trial. Lancet Neurol.

[24] Edaravone Acute Infarction Study G (2003). Effect of a novel free radical scavenger, edaravone (MCI-186), on acute brain infarction. Randomized, placebo-controlled, double-blind study at multicenters. Cerebrovasc Dis.

[25] Watanabe K, Tanaka M, Yuki S, Hirai M, Yamamoto Y (2018). How is edaravone effective against acute ischemic stroke and amyotrophic lateral sclerosis?. J Clin Biochem Nutr.

[26] Yang R, Wang Q, Li F, Li J, Liu X (2015). Edaravone injection ameliorates cognitive deficits in rat model of Alzheimer’s disease. Neurol Sci.

[27] Liu R, Zhang L, Lan X, Li L, Zhang TT, Sun JH, Du GH (2011). Protection by borneol on cortical neurons against oxygen-glucose deprivation/reperfusion: involvement of anti-oxidation and anti-inflammation through nuclear transcription factor kappaappaB signaling pathway. Neuroscience.

[28] Almeida JR, Souza GR, Silva JC, Saraiva SR, Junior RG, Quintans Jde S, Barreto Rde S, Bonjardim LR, Cavalcanti SC, Quintans LJ Jr. Borneol, a bicyclic monoterpene alcohol, reduces nociceptive behavior and inflammatory response in mice. *ScientificWorldJournal* 2013, 2013:808460. 10.1155/2013/808460 PMC3654274 23710149

[29] Wu T, Zhang A, Lu H, Cheng Q (2018). The role and mechanism of Borneol to open the blood-brain barrier. Integr Cancer Ther.

[30] Hua Y, Zhou L, Yang W, An W, Kou X, Ren J, Su H, Chen R, Zhang Z, Zou J, Zhao Z (2021). Y-2 reduces oxidative stress and inflammation and improves neurological function of collagenase-induced intracerebral hemorrhage rats. Eur J Pharmacol.

[31] Xu J, Wang A, Meng X, Yalkun G, Xu A, Gao Z, Chen H, Ji Y, Xu J, Geng D (2021). Edaravone Dexborneol Versus Edaravone alone for the treatment of Acute ischemic stroke: a phase III, Randomized, Double-Blind, comparative trial. Stroke.

[32] Bundy JL, Vied C, Badger C, Nowakowski RS (2019). Sex-biased hippocampal pathology in the 5XFAD mouse model of Alzheimer’s disease: a multi-omic analysis. J Comp Neurol.

[33] Sil A, Erfani A, Lamb N, Copland R, Riedel G, Platt B (2022). Sex differences in Behavior and Molecular Pathology in the 5XFAD model. J Alzheimers Dis.

[34] Oakley H, Cole SL, Logan S, Maus E, Shao P, Craft J, Guillozet-Bongaarts A, Ohno M, Disterhoft J, Van Eldik L (2006). Intraneuronal beta-amyloid aggregates, neurodegeneration, and neuron loss in transgenic mice with five familial Alzheimer’s disease mutations: potential factors in amyloid plaque formation. J Neurosci.

[35] Richetin K, Steullet P, Pachoud M, Perbet R, Parietti E, Maheswaran M, Eddarkaoui S, Begard S, Pythoud C, Rey M (2020). Tau accumulation in astrocytes of the dentate gyrus induces neuronal dysfunction and memory deficits in Alzheimer’s disease. Nat Neurosci.

[36] Jeon SG, Lee HJ, Park H, Han KM, Hoe HS (2020). The VEGF inhibitor vatalanib regulates AD pathology in 5xFAD mice. Mol Brain.

[37] Caccamo A, Branca C, Piras IS, Ferreira E, Huentelman MJ, Liang WS, Readhead B, Dudley JT, Spangenberg EE, Green KN (2017). Necroptosis activation in Alzheimer’s disease. Nat Neurosci.

[38] Kwon HS, Koh SH (2020). Neuroinflammation in neurodegenerative disorders: the roles of microglia and astrocytes. Transl Neurodegener.

[39] Hall G, Singh IS, Hester L, Hasday JD, Rogers TB (2005). Inhibitor-kappab kinase-beta regulates LPS-induced TNF-alpha production in cardiac myocytes through modulation of NF-kappaB p65 subunit phosphorylation. Am J Physiol Heart Circ Physiol.

[40] Boissiere F, Hunot S, Faucheux B, Duyckaerts C, Hauw JJ, Agid Y, Hirsch EC (1997). Nuclear translocation of NF-kappaB in cholinergic neurons of patients with Alzheimer’s disease. NeuroReport.

[41] Braidy N, Guillemin G, Grant R (2008). Promotion of cellular NAD(+) anabolism: therapeutic potential for oxidative stress in ageing and Alzheimer’s disease. Neurotox Res.

[42] Khakh BS, Deneen B (2019). The Emerging Nature of Astrocyte Diversity. Annu Rev Neurosci.

[43] Re DB, Le Verche V, Yu C, Amoroso MW, Politi KA, Phani S, Ikiz B, Hoffmann L, Koolen M, Nagata T (2014). Necroptosis drives motor neuron death in models of both sporadic and familial ALS. Neuron.

[44] Zelic M, Pontarelli F, Woodworth L, Zhu C, Mahan A, Ren Y, LaMorte M, Gruber R, Keane A, Loring P (2021). RIPK1 activation mediates neuroinflammation and disease progression in multiple sclerosis. Cell Rep.

[45] White JA, Manelli AM, Holmberg KH, Van Eldik LJ, Ladu MJ (2005). Differential effects of oligomeric and fibrillar amyloid-beta 1–42 on astrocyte-mediated inflammation. Neurobiol Dis.

[46] Chang L, Yin CY, Wu HY, Tian BB, Zhu Y, Luo CX, Zhu DY (2017). (+)-Borneol is neuroprotective against permanent cerebral ischemia in rats by suppressing production of proinflammatory cytokines. J Biomed Res.

[47] Cummings J, Lee G, Nahed P, Kambar M, Zhong K, Fonseca J, Taghva K (2022). Alzheimer’s disease drug development pipeline: 2022. Alzheimers Dement (N Y).

[48] Dehghan E, Zhang Y, Saremi B, Yadavali S, Hakimi A, Dehghani M, Goodarzi M, Tu X, Robertson S, Lin R (2017). Hydralazine induces stress resistance and extends C. Elegans lifespan by activating the NRF2/SKN-1 signalling pathway. Nat Commun.

[49] Fotuhi M, Mohassel P, Yaffe K (2009). Fish consumption, long-chain omega-3 fatty acids and risk of cognitive decline or Alzheimer disease: a complex association. Nat Clin Pract Neurol.

[50] Chiu CC, Su KP, Cheng TC, Liu HC, Chang CJ, Dewey ME, Stewart R, Huang SY (2008). The effects of omega-3 fatty acids monotherapy in Alzheimer’s disease and mild cognitive impairment: a preliminary randomized double-blind placebo-controlled study. Prog Neuropsychopharmacol Biol Psychiatry.

[51] Xu J, Wang Y, Wang A, Gao Z, Gao X, Chen H, Zhou J, Zhao X, Wang Y (2019). Safety and efficacy of Edaravone Dexborneol versus edaravone for patients with acute ischaemic stroke: a phase II, multicentre, randomised, double-blind, multiple-dose, active-controlled clinical trial. Stroke Vasc Neurol.

[52] Kraeuter AK, Guest PC, Sarnyai Z (2019). The Y-Maze for Assessment of spatial working and reference memory in mice. Methods Mol Biol.

[53] Sima J, Yan Z, Chen Y, Lehrmann E, Zhang Y, Nagaraja R, Wang W, Wang Z, Schlessinger D (2018). Eda-activated RelB recruits an SWI/SNF (BAF) chromatin-remodeling complex and initiates gene transcription in skin appendage formation. Proc Natl Acad Sci U S A.

[54] Stine WB, Jungbauer L, Yu C, LaDu MJ (2011). Preparing synthetic abeta in different aggregation states. Methods Mol Biol.

