# Supplementary material for: Edaravone Dexborneol mitigates pathology in animal and cell culture models of Alzheimer’s disease by inhibiting neuroinflammation and neuronal necroptosis
Source: Cell Biosci. 2024 Apr 27;14:55. doi: 10.1186/s13578-024-01230-8 (PMC11056062; doi:10.1186/s13578-024-01230-8)
Supplement: Supplementary file 1 — Supplementary Material 1 [file 13578_2024_1230_MOESM1_ESM.docx]

**Supplementary Information**

**Edaravone Dexborneol mitigates pathology in animal and cell culture models of Alzheimer's disease by inhibiting neuroinflammation and neuronal necroptosis**

Chong Xu^1^, Yilan Mei^1^, Ruihan Yang^1^, Qiudan Luo^1^, Jienian Zhang^1^, Xiaolin Kou^2^, Jianfeng Hu^1,2^, Yujie Wang^1^, Yue Li^1^, Rong Chen^2^, Zhengping Zhang^2^*, Yuyuan Yao^1^* and Jian Sima^1^*

1. Laboratory of Aging Neuroscience and Neuropharmacology, School of Basic Medicine and Clinical Pharmacy, China Pharmaceutical University, Nanjing, 210009, China.
2. Department of Pharmacology, NeuroDawn Pharmaceutical Co., Ltd., Nanjing, 211199, China.

* To whom correspondence should be addressed.

Jian Sima, Email: simajian@cpu.edu.cn

Yuyuan Yao, Email: yuyuanyao@cpu.edu.cn

Zhengping Zhang, Email: zhangzhengping@neurodawn.cn


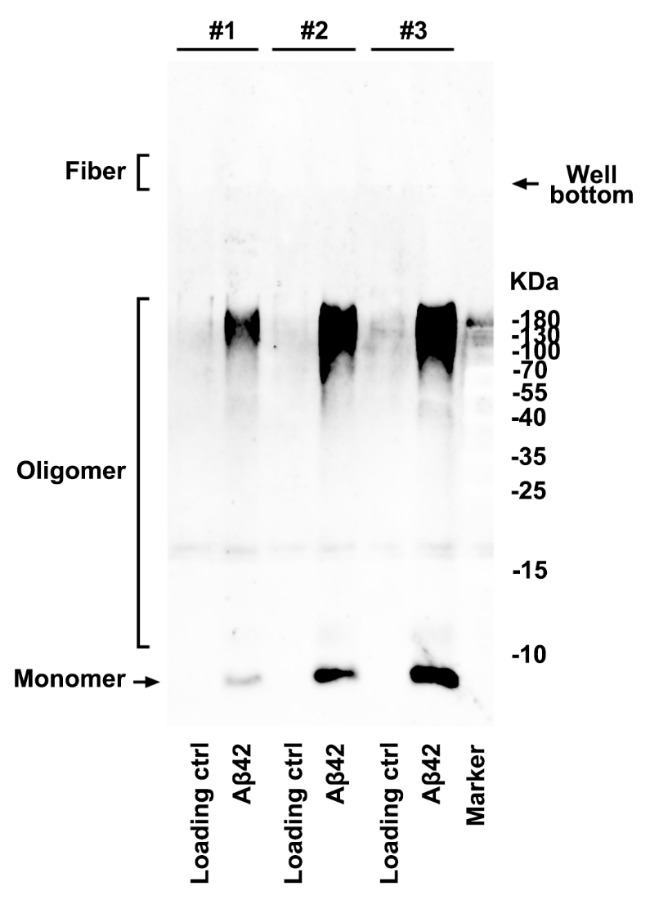


**Figure S1 The validation of Aβ Oligomerization**

Immunoblotting shows the feature of Aβ oligomers using an anti-β-Amyloid antibody (Cell Signaling Technology, #2454S, 1:1000 dilution). This polyclonal antibody was produced by immunizing animals with a synthetic peptide corresponding to residues at the amino terminus of human β-amyloid peptides).


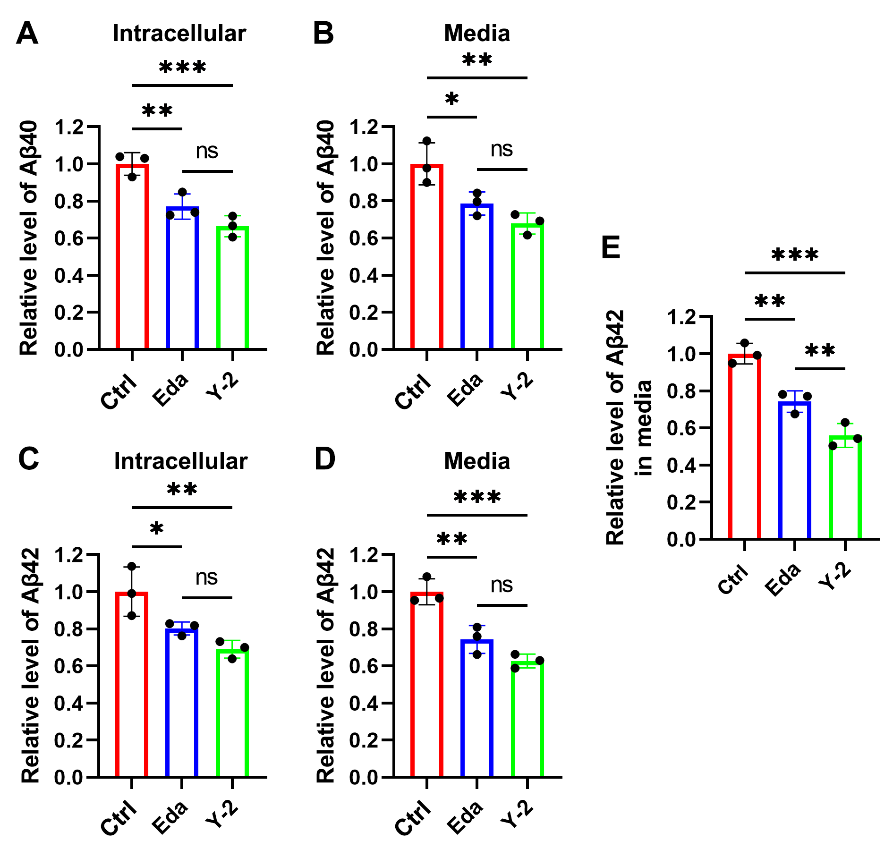


**Figure S2 Y-2 inhibits Aβ production and promotes Aβ clearance**

(A-D) Quantification of intracellular and extracellular (culture media) Aβ40 and Aβ42 produced by APP695-SH-SY5Y cells treated with Eda or Y-2. (E) Quantification of residual Aβ42 in the media following the clearance by BV-2 cells pre-treated with Eda or Y-2, after exposure to soluble Aβ42 for 12 h. n=3. The level of Aβ in the Ctrl group was normalized to 1.0. n represents the number of independent experiments. One-way ANOVA with Fisher’s LSD post hoc analysis; **P*<0.05, ***P*<0.01, ****P*<0.001. All data are represented as mean ± SD.

**
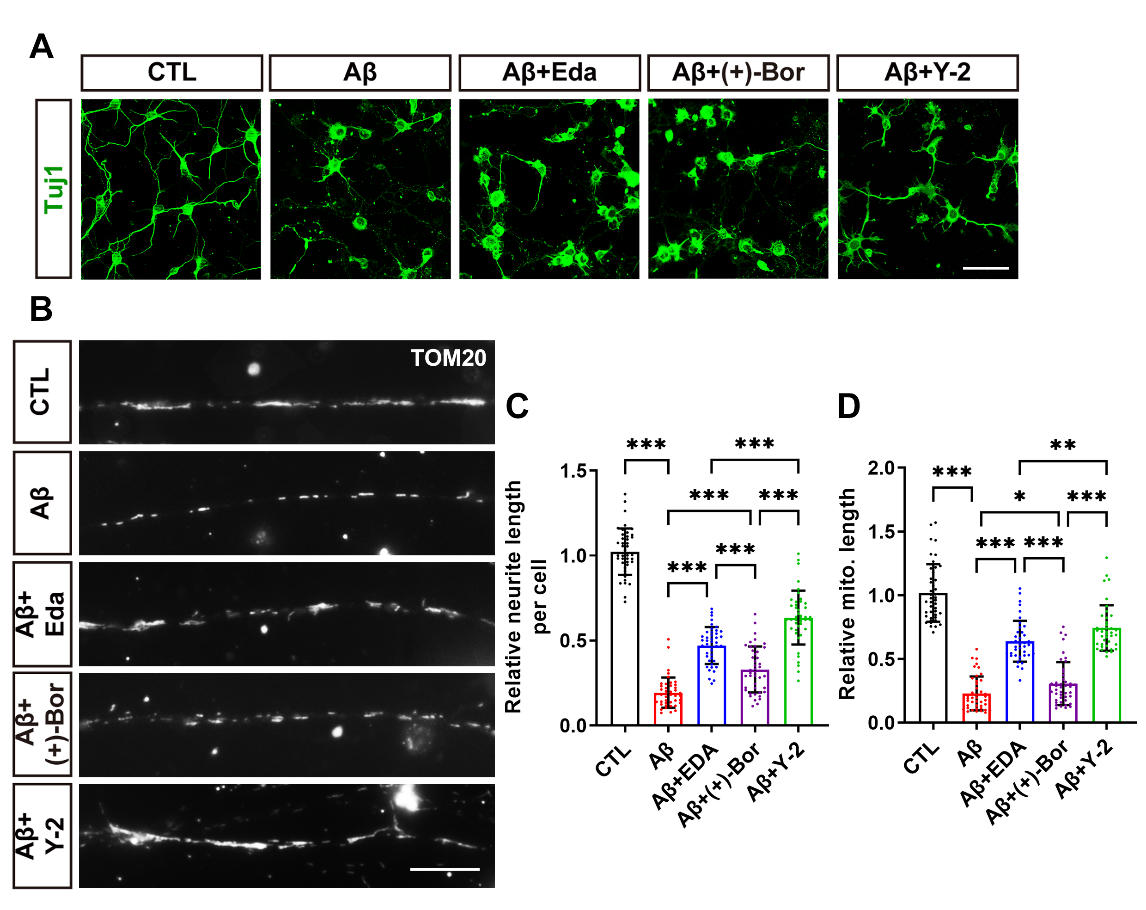
**

**Figure S3 Y-2 inhibits Aβ-induced neurite injury and mitochondrial fragmentation in primary neurons**

(A) IF of neuronal marker Tuj1 (green) in primary cortical neurons with indicated treatments. Scale bar, 50 μm. (B) IF of TOM20 shows the mitochondrial segments in primary neurons with indicated treatments. Scale bar, 10 μm. (C) Quantification of neurite length in (A), n≥40. n represents the number of random images from 3 independent experiments. one-way ANOVA with Fisher’s LSD post hoc analysis; ****P*<0.001. The total neurite length per cell in CTL group was normalized to 1.0. (D) The quantification shows the length of mitochondrial (mito.) segments in (B), n≥20. n represents the number of randomly selected neurites from 3 independent experiments. one-way ANOVA with Fisher’s LSD post hoc analysis; **P*<0.05, ***P*<0.01, ****P*<0.001. The mito. length in CTL group was normalized to 1.0. All data are represented as mean ± SD.

**
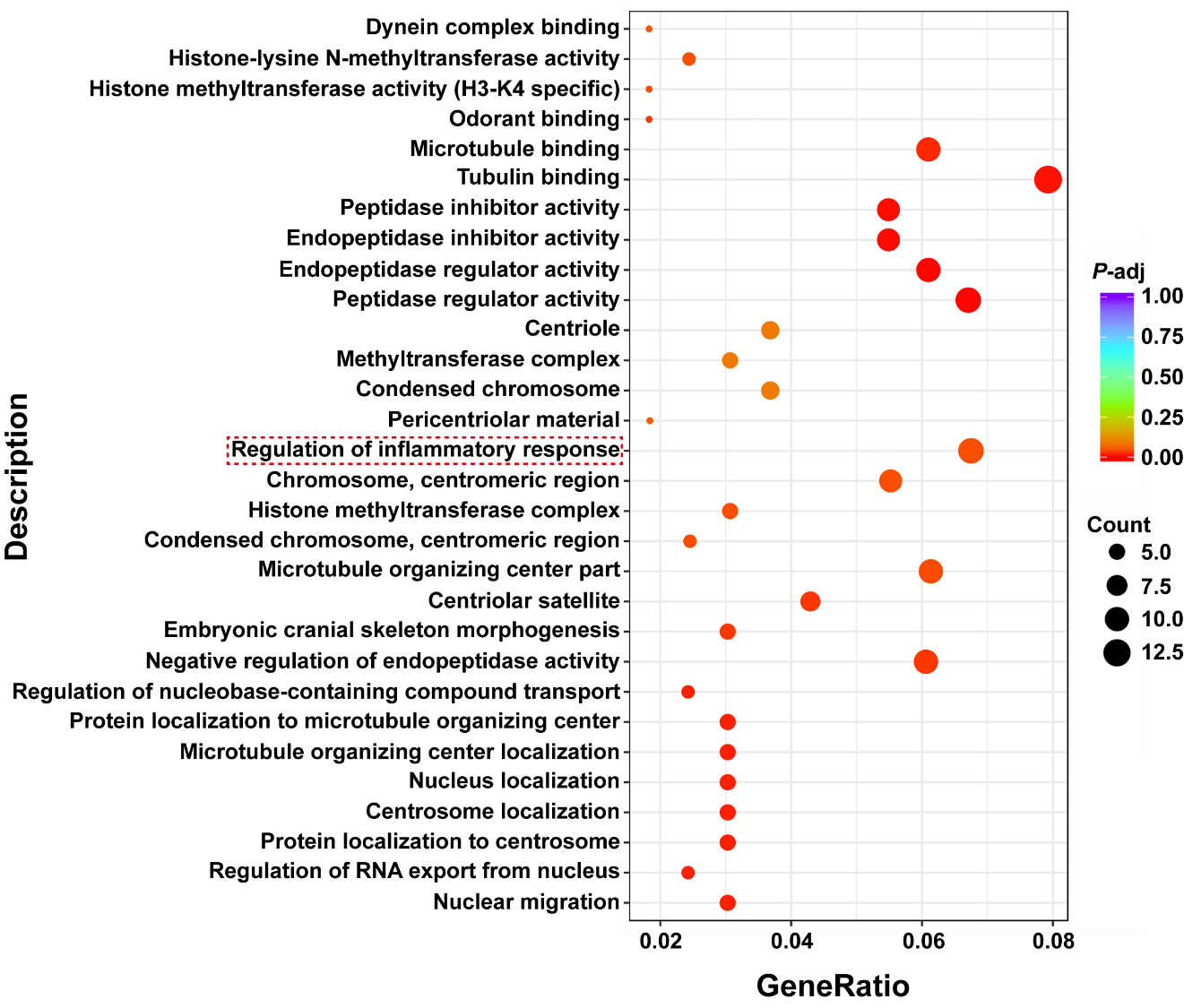
**

**Figure S4 The Gene Ontology (GO) analysis [(+)-Borneol versus Vehicle] in Aβ-treated astrocytes**

Gene Ontology (GO) enrichment analysis [Aβ+(+)-Borneol versus Aβ+Vehicle]. The vertical coordinates are the enriched pathways, and the horizontal coordinates are the gene ratio. The size of each point represents the number of downregulated genes in the pathway, and the color of the point represents the *P*-adjust.


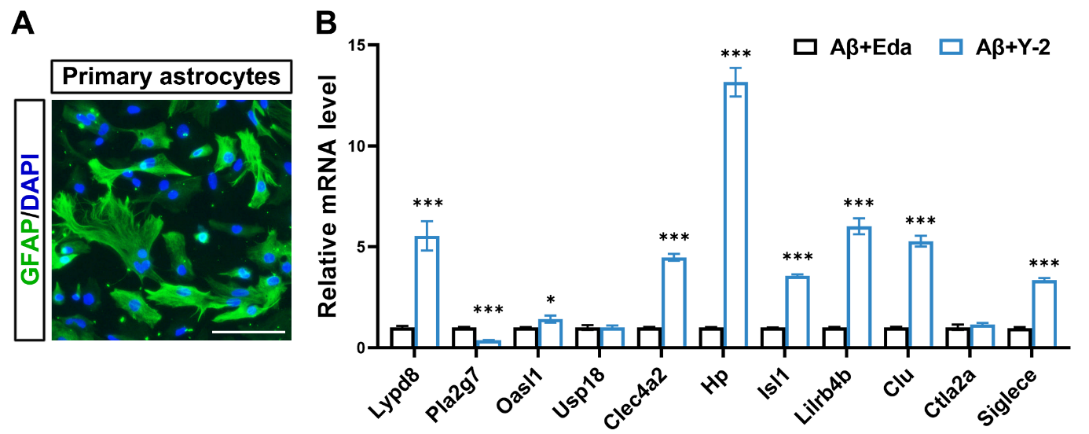


**Figure S5 mRNA levels in primary astrocytes**

(A) The primary mouse astrocytes identified by GFAP IF. Scale bar, 50 μm. (B) mRNA levels of representative genes in primary astrocytes are shown by real-time qPCR analyses, n=3. n represents the number of independent experiments. **P*<0.05, ****P*<0.001; Student’s *t*-test. All data are represented as mean ± SD.


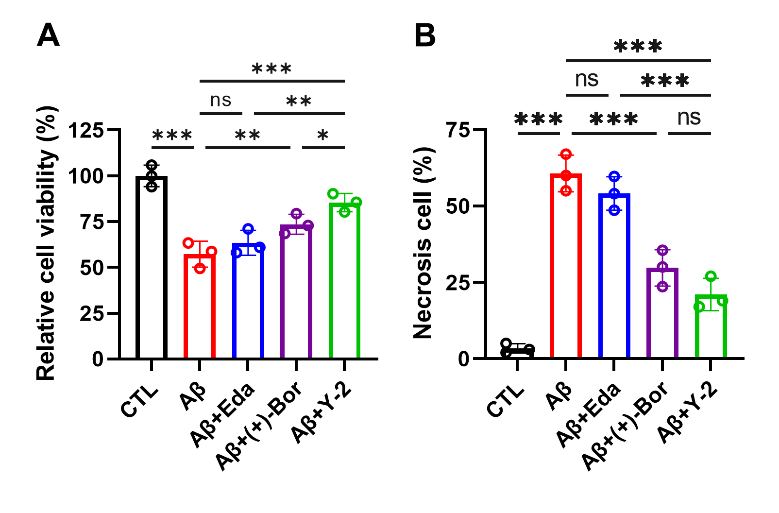


**Figure S6 Supplementary information related to Figure 7**

(A and B) Quantitation of cell viability (A) and necrotic cell (B) of SH-SY5Y cells treated with conditioned media from astrocytic C8-D1A cultures with indicated treatments, n=3. n represents the number of independent experiments. The cell viability in CTL group was normalized to 100%. One-way ANOVA with Fisher’s LSD post hoc analysis; **P*<0.05, ***P*<0.01, ****P*<0.001. All data are represented as mean ± SD.

**Supplementary Table 1 qPCR primers used in this study**

| **Primer name** | **DNA sequence (5’-3’)** |
| --- | --- |
| Mouse Duoxa1-F | ACCAAGCCAACCTTTCCAATG |
| Mouse Duoxa1-R | GCCCCGATGAATAAGCTGGTC |
| Mouse Isl1-F | ATGATGGTGGTTTACAGGCTAAC |
| Mouse Isl1-R | TCGATGCTACTTCACTGCCAG |
| Mouse Usp18-F | TTGGGCTCCTGAGGAAACC |
| Mouse Usp18-R | CGATGTTGTGTAAACCAACCAGA |
| Mouse Ctla2a-F | CTCCACCCCCTGATCCAAGT |
| Mouse Ctla2a-R | ACACGAGTCTTCTGTGTCTTTCT |
| Mouse Lilrb4b-F | AGTGTCGTCACAAAAATAAGGCT |
| Mouse Lilrb4b-R | CCTGGGCGTACACAATTCCC |
| Mouse Lpl-F | GGGAGTTTGGCTCCAGAGTTT |
| Mouse Lpl-R | TGTGTCTTCAGGGGTCCTTAG |
| Mouse Siglece-F | GGAGGGTCAGAACCCCCAA |
| Mouse Siglece-R | TGAGATAGGAGAAGTTACAGGGC |
| Mouse Pla2g7-F | CTTTTCACTGGCAAGACACATCT |
| Mouse Pla2g7-R | CGACGGGGTACGATCCATTTC |
| Mouse Nt5dc1-F | GCTTCGATCTCGACCACACG |
| Mouse Nt5dc1-R | CTGCAAGTTTAATGAAGGTCCCA |
| Mouse Clu-F | AGCAGGAGGTCTCTGACAATG |
| Mouse Clu-R | GGCTTCCTCTAAACTGTTGAGC |
| Mouse Vsir-F | GGAACCCTGCTCCTTGCTATT |
| Mouse Vsir-R | TTGTAGATGGTCACATCGTGC |
| Mouse Clec4a2-F | CCCCCATTGGACAAAGGGC |
| Mouse Clec4a2-R | GGTGCCAAGATACCCAAGTCTA |
| Mouse Hp-F | GCTATGTGGAGCACTTGGTTC |
| Mouse Hp-R | CACCCATTGCTTCTCGTCGTT |
| Mouse Lyz1-F | GAGACCGAAGCACCGACTATG |
| Mouse Lyz1-R | CGGTTTTGACATTGTGTTCGC |
| Mouse Oasl1-F | CAGGAGCTGTACGGCTTCC |
| Mouse Oasl1-R | CCTACCTTGAGTACCTTGAGCAC |
| Mouse Lypd8-F | TCTGCTCCGCATCGAATTG |
| Mouse Lypd8-R | ACCATAGCAAGACATGCACTG |
| Mouse Hba-a1-F | CACCACCAAGACCTACTTTCC |
| Mouse Hba-a1-R | CAGTGGCTCAGGAGCTTGA |
